# Prediction Models for Osteoporotic Fractures Risk: A Systematic Review and Critical Appraisal

**DOI:** 10.14336/AD.2021.1206

**Published:** 2022-07-11

**Authors:** Xuemei Sun, Yancong Chen, Yinyan Gao, Zixuan Zhang, Lang Qin, Jinlu Song, Huan Wang, Irene XY Wu

**Affiliations:** ^1^Department of Epidemiology and Biostatistics, Xiangya School of Public Health, Central South University, Changsha 410000, Hunan, China.; ^2^Hunan Provincial Key Laboratory of Clinical Epidemiology, Changsha 410000, China

**Keywords:** osteoporotic fractures, prediction model, systematic review, critical appraisal

## Abstract

Osteoporotic fractures (OF) are a global public health problem currently. Many risk prediction models for OF have been developed, but their performance and methodological quality are unclear. We conducted this systematic review to summarize and critically appraise the OF risk prediction models. Three databases were searched until April 2021. Studies developing or validating multivariable models for OF risk prediction were considered eligible. Used the prediction model risk of bias assessment tool to appraise the risk of bias and applicability of included models. All results were narratively summarized and described. A total of 68 studies describing 70 newly developed prediction models and 138 external validations were included. Most models were explicitly developed (n=31, 44%) and validated (n=76, 55%) only for female. Only 22 developed models (31%) were externally validated. The most validated tool was Fracture Risk Assessment Tool. Overall, only a few models showed outstanding (n=3, 1%) or excellent (n=32, 15%) prediction discrimination. Calibration of developed models (n=25, 36%) or external validation models (n=33, 24%) were rarely assessed. No model was rated as low risk of bias, mostly because of an insufficient number of cases and inappropriate assessment of calibration. There are a certain number of OF risk prediction models. However, few models have been thoroughly internally validated or externally validated (with calibration being unassessed for most of the models), and all models showed methodological shortcomings. Instead of developing completely new models, future research is suggested to validate, improve, and analyze the impact of existing models.

Osteoporotic fractures (OF) are fractures that occur during minor trauma or daily activities, which are a serious consequence of osteoporosis [1]. The common fracture sites are vertebral, hip, distal radius, proximal humerus, and pelvis [2]. Osteoporosis causes more than nine million new fractures worldwide every year, it is estimated that an OF occurs every three seconds [3], and one-third of women and one-fifth of men will suffer an OF in their lifetime [4]. OF can cause pain, severe disability and mortality, as well as burdens on families and society. It seriously impairs the quality of life of patients [5].

Prevention of OF requires early and accurate identification of individuals at risk and taking effective preventive interventions in time [6]. Bone mineral density (BMD) test is the gold standard for diagnosing osteoporosis. It is often used to identify patients with osteoporosis or low BMD. Nevertheless, studies have shown that the BMD test alone does not reliably predict whether individuals will develop a fracture [7]. In addition, high cost, ionizing radiation, and low mobility of the BMD test limit its clinical application [8]. Therefore, in many clinical guidelines, it is now recommended to use prediction models integrating several risk factors to identify individuals at high risk of OF [9].

At present, numerous prediction tools for OF have been developed, including but not limited to the World Health Organization (WHO) Fracture Risk Assessment Tool (FRAX) algorithm [10], Qfracture algorithm [11], and Garvan Fracture Risk Calculator (Garvan) [12]. Some of them have been recommended in clinical guidelines for treatment management [13,14] and more and more advocated by health policymakers. Although there are some systematic reviews on OF prediction models [15-17], they are outdated with the latest literature search being performed in 2017 [16]. Further limitations include restriction to a few specific tools [17] or a certain population like women [15], or no critical appraisal of the included models with standardized criteria [16,17]. Hence, an updated systematic review of prediction models for OF is needed.

We conducted this systematic review and critical appraisal to summarize the characteristics of the development and validation of OF risk prediction model, assess its methodological quality and reporting quality, and provide up-to-date evidence for clinical implementation and future research.

## METHODS

This systematic review was reported by following the Preferred Reporting Items for Systematic Reviews and Meta-Analyses (PRISMA) [18]. The protocol of this systematic review has been registered in PROSPERO (registration number: CRD42020199196).

### Search strategy

We systematically searched PubMed, Embase, and PsycINFO from inception to April 3, 2021. In addition, the reference lists of included studies were manually reviewed. The search strategy included the key concepts of i) osteoporotic fractures and osteoporosis and ii) risk prediction and related terms. The detailed search strategies are presented in Supplementary table 1.

### Eligible criteria

Cohort studies that develop or validate risk prediction models for OF in the general population were considered eligible. Studies were excluded if i) the prediction model consisted of only one predictor; ii) they targeted secondary OF or focused on specific patient groups for the treatment of OF or related conditions; iii) the performance of the model was not reported; iv) they were reviews, conference abstracts, letters or protocols. In addition, if the development article was not available, the corresponding externally verified articles were excluded.

### Literature selection

Two reviewers (HW and JS) independently selected the studies, determined eligibility, and resolved the discrepancies by consensus. When the difference is not resolved, the third reviewer (LQ) was invited to make a consensus decision.

### Data extraction

Two reviewers (XS and YC) independently extracted the data with a pre-developed data extraction form, which was developed by following the guidance of the critical appraisal and data extraction for systematic reviews of prediction modelling studies (CHARMS) checklist [19]. Extracted the following information from each included study: i) characteristics of the study (e.g., study design, data source); ii) data related to participants (e.g., country or region of participants, age, gender, events per variable (EPV)); iii) details about model development and validation (e.g., type of prediction model, predictors included in the model, modelling method) and model performance.

Multiple different models were included in a study, for example, separate models for men and women, separate models for different outcomes (e.g., hip fracture, major osteoporotic fractures (MOF), were included separately. When multiple versions (e.g., with different risk factors) of a model for the same population and outcome were included in a study, the model with the best performance was selected for data extraction. When an article validated multiple models, separate data extraction was performed for each model.

Model performance was assessed by discrimination and calibration. Discrimination is often quantified by the C index or area under the receiver operating characteristic curve (AUC). A C index or AUC less than 0.5 suggests no discrimination, 0.5 to 0.7 is poor, 0.7 to 0.8 is acceptable, 0.8 to 0.9 is excellent, and higher than 0.9 is outstanding [20]. Calibration can be visualized by a calibration plot and is usually quantified using the calibration intercept and the calibration slope, with a slope close to 1 and an intercept close to 0 indicating good calibration [21]. The indexes mentioned above were extracted from the publications when available. Sensitivity and specificity were extracted as well if available. Additionally, EPV was calculated to measure model overfitting. An EPV less than 20 was considered as overfitting for model development while less than 100 for model validation [22].

### Risk of bias and applicability assessment

The risk of bias and applicability of each included study was independently assessed by two reviewers (ZZ and XS) using the prediction model risk of bias assessment tool (PROBAST) [23,24]. Discrepancies were resolved by consensus between the two reviewers, and a third author (YG) was invited for consensus adjudication in need. For risk of bias assessment, it contains four domains: participants, predictors, outcome, and analysis. Each domain was judged as low, high, or unclear risk of bias. The overall risk of bias was summarized according to the following rules: when all the four domains were judged as “low” risk of bias, the overall risk of bias was “low”; otherwise, “high” or “unclear” risk of bias was graded accordingly [23,24]. For applicability assessment, it contains three domains: participants, predictors, and outcome. It has similar assessment rules and procedures to the risk of bias assessment.

### Statistical Analysis

All results were narratively summarized and described without any quantitative synthesis due to variation in predictors and characteristics of participants among the included prediction models.

**Figure 1.**
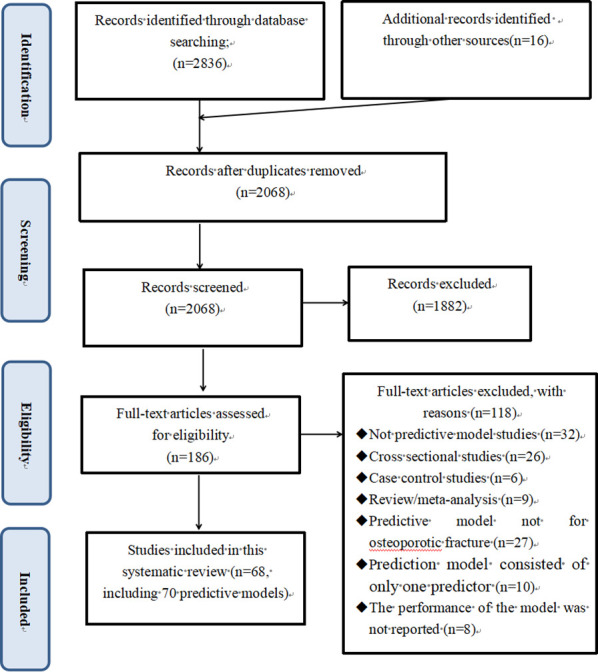
PRISMA flow diagram for literature search and selection.

## RESULTS

### Study selection

The literature search identified 2852 records, of which 784 were removed due to duplication, and 1882 were excluded based on title and abstract. A total of 186 full texts were assessed, of which 68 articles met the eligibility criteria were included in this review (Fig. 1). In total, 38 articles focused on one or more development of OF risk prediction models, and 44 articles described one or more external validation of OF risk prediction models. Articles frequently concern combinations of development and external validation, leading to the total number of articles does not sum up to 68.

### Studies focused on the development of OF prediction models

#### Populations and outcomes

Thirty-eight [10-12,25-59] articles represented the development of 70 different models in total. Most of the participants were from the UK (n=17, 24%), China (n=17, 24%) or the US (n=12, 17%), the remaining (n=24, 34%) were from other countries in Oceania, Western Europe, or East Asia, while there were no models developed using data from Africa, South America, and the Middle East. The average age of participants ranged from 56.7 to 80.5 years. The follow-up duration ranged from 1 to 13 years, with 30 (43%) models equal to or more than 10 years. The outcomes covered MOF (n=35, 50%), hip fracture (n=25, 36%), any fractures (n=6, 9%), and other fractures (n=4, 6%). Diagnosis of fracture was mostly through medical records (n=39, 56%) or self-reported (n=18, 26%), 11 (16%) models were radiographic reports, and the remaining two (3%) models were self-reported and confirmed by medical records (Table 1).

**Table 1:** Basic characteristics of included studies.

First author, year	Model; No.^a^	Study design	Data source; country or region of participants	Age (SD) (years)	Female (%)	Follow up duration (SD) (year)	Outcome	Measurement of fracture	Incidence of fracture (%)	Sample size
**Model development^b^**										
Dargent- Molina 2002^[25]^	NR; 1	R	EPIDemiologie de l'OSteoporose study; France	80.5(3.7)	100	3.7(0.8)	Hip fracture	Self-reported	4.0	6933
Colón- Emeric 2002^l^ ^[26]^	NR; 4	R	Established population for epidemiologic studies of the elderly; US	M: 73.4(6.7) F: 74.5(6.6)	65.0	3.0(NR)	Any fractures^c^	Self-reported	Hip: 3.8 Any: 11.0	7654
McGrother 2002^[27]^	NR; 1	P	A large general practice; UK	77.9(6.1)	100	3.0(NR)	Hip fracture	Medical records	2.0	1289
Albertsson 2007^[28]^	FRAMO; 1	R	Three rural primary health care; Sweden	78.8(6.5)	100	2.0(NR)	Hip fracture	Radiographic reports	1.2	1248
Robbins 2007^[29]^	WHI; 1	P	Female’s Health Initiative 40 clinical centers; US	NR	100	8.0(1.7)	Hip fracture	Self-reported and confirmed by medical records	0.1	93676
Nguyen 2008^[12]^	Garvan; 4	P	Dubbo osteoporosis epidemiology study; Australia	M: 70.0(6.0) F: 71.0(8.0)	61.3	M: 12.0(NR) F: 13.0(NR)	Any fractures	Radiographic reports	M: 17.4 F: 31.4	M: 858 F: 1358
Kanis 2008^l^ ^[10]^	FRAX; 8	P	Nine population-based cohort studies^d^; UK	65.0	68.0	10.0(NR)	MOF^e^	Self-reported or confirmed by medical records	Hip: 1.8 MOF: 7.2	273826
Hippisley- Cox 2009^[11]^	QFracture; 4	P	Version 20 of the QResearch; UK	NR	NR	10.0(NR)	MOF	Medical records	Hip: 0.4(M) 1.2(F) MOF: 1.0(M) 3.1(F)	M: 1807996 F: 1825816
Tanaka 2010^l^ ^[30]^	FRISC; 2	R	Three population-based cohort studies; Japan	63.4(11.1)	100	5.3(NR)	Any fractures^f^	Medical records	21.4	2187
Yun 2010^k^ ^[31]^	NR, FRAX; 4	R	Medicare current beneficiary survey; UK	NR	NR	2.0(NR)	MOF	Medical records	NR	12337
Sambrook 2011^[32]^	NR; 1	R	The global longitudinal Osteoporosis study^g^; UK	NR	100	2.0(NR)	Hip fracture	Self-reported	4.5	19586
Bow 2011^[33]^	NR; 1	P	Mr. and Ms. Os study; China	68.0(10.3)	0	3.5(2.9)	MOF	Self-reported and confirmed by medical records	2.0	1,810
Henry 2011^k^ ^[34]^	FRISK, FRAX, Garvan; 4	P	Geelong osteoporosis study; Australia	NR	100	9.6(NR)	MOF	Radiographic reports	20.8	600
Tamaki 2011^k^ ^[35]^	NR, FRAX; 6	R	Population-based cohort study; Japan	56.7(9.6)	100	10.0(NR)	MOF	Radiographic reports	MOF: 5.3 Hip: 0.5	815
Hippisley- Cox 2012^[36]^	Updated QFracture; 4	P	Version 32 of the QResearch; UK	NR	NR	10.0(NR)	MOF	Medical records	Hip: 0.3(M) 0.9(F) MOF: 0.9(M) 2.8(F)	4726046
LaFleur 2012^[37]^	NR; 2	P	Veterans health administration system; US	66.9(10.3)	0	2.8(NR)	MOF	Medical records	Hip: 0.3 MOF: 1.2	84763
Schousboe 2014^[38]^	NR; 1	P	Study of osteoporotic Fractures; US	75.0	100	NR	Vertebral fractures	Radiographic reports	20.4	5560
Yu 2014^k^ ^[39]^	FRAX+S, FRAX; 16	P	Population-based cohort study; China	72.5(5.2)	50.0	10.2	MOF	Medical records	Hip: 3.3 MOF: 14.1	4000
Iki 2015^k^ ^[40]^	FRAX +TBS, FRAX; 2	P	Study of Fujiwara-kyo Osteoporosis Risk in male; Japan	73.0(5.1)	0	4.5(NR)	MOF	Radiographic reports	1.2	1872
Jang 2016^[41]^	NR; 2	P	Health and genome study; Korea	M: 61.3(7.1) F: 61.1(7.1)	52.7	7.0(NR)	MOF	Self-reported	M:9.9 F: 12.3	M: 363 F: 405
Kim 2016^[42]^	KFRS; 2	P	National Health Insurance Service; Korea	M: 59.8(7.9) F: 60.6(8.3)	48.5	7.0(NR)	MOF	Medical records	M: 1.3 F: 4.3	M: 370225 F: 348253
Francesco 2017^l^ ^[43]^	FRA-HS; 2	P	IMS health longitudinal study; Italy	60.1(12.8)	55.0	10.0(NR)	MOF	Medical records	5.9	490013
Kruse 2017^[44]^	NR; 2	R	Health database; Denmark	NR	86.1	5.0(NR)	Hip, femoral fractures	Medical records	6.6(M/F)	M: 717 F: 4722
Li 2017^[45]^	NR; 1	P	Global longitudinal study of osteoporosis in female 3-year cohort; Canada	69.4(8.9)	100	3.0(NR)	MOF	Self-reported	4.0	3985
Su 2017^[46]^	NR; 2	P	Mr. and Ms. Os study; China	M: 72.4(NR) F: 72.6(NR)	50.3	M: 9.9(2.8) F: 8.8(1.5)	MOF	Medical records	M: 6.6 F: 11.0	M: 1923 F: 1950
Weycker 2017^[47]^	NR; 2	R	Study of osteoporotic fractures; US	NR	100	1.0(NR)	Any fractures^h^	Self-reported	Hip: 2.2 Non vertebral: 6.6	2,499
Sundh 2017^k^ ^[48]^	FRAX+MS, FRAX; 2	P	Population-based cohort study; Sweden	NR	100	10.0(NR)	MOF	Medical records	16.3	412
Biver 2018^k^ ^[50]^	NR, FRAX; 2	P	Geneva retirees cohort study; Switzerland	65.0(1.4)	100	5.0(1.8)	MOF	Self-reported	19.1	740
Reber 2018^[49]^	NR; 1	R	Social insurance for agriculture, forestry and horticulture; Germany	75.4(6.3)	48.8	2.0(NR)	MOF	Medical records	2.6	298530
Su 2018^k^ ^[52]^	FRAX+Fall, FRAX; 4	P	Mr. and Ms. Os study; China	M: 72.4(NR) F: 72.6(NR)	50.0	M: 9.9(2.8) F: 8.8(1.5)	MOF	Medical records	M: 7.0 F: 11.8	M: 2000 F: 2000
Rubin 2018^[51]^	FREM; 2	P	National registers data; Denmark	NR	51.9	10.0(NR)	MOF	Medical records	M: 0.6 F: 1.4	M: 12011143 F: 1294206
Su 2019(1)^k^ ^[53]^	NR, FRAX; 3	P	Osteoporotic fractures in men; China	73.6(5.9)	0	8.6(2.5)	Hip fracture	Self-reported or confirmed by radiographic reports	2.9	5977
Engels 2020^[54]^	NR; 1	R	Administrative claims data; Germany	75.7(6.20)	48.8	4.0(NR)	Hip fracture	Medical records	0.6	78074
Kong 2020^[55]^	NR; 1	P	Health and genome Study; Korea	61.2(8.7)	56.4	7.5(1.6)	MOF	Self-reported or confirmed by radiographic reports	25.6	2227
Sheer 2020^[56]^	NR; 1	R	Humana research; US	74.3(NR)	56.0	1.0(NR)	MOF	Medical records or self-reported	6.6	1287354
Wu 2020^[57]^	NR; 1	P	Osteoporotic fractures in men Study; US	NR	0	NR	MOF	Radiographic reports	8.8	5130
Lu 2021^m^ ^[58]^	GSOS, FRAX; 6	R	Five population-based cohort studies^i^; UK, US, Sweden, China	NR	54.0	NR	MOF	Medical records or radiographic reports	Hip: 2.5 MOF: 6.0	431621
de Vries 2021^[59]^	NR; 1	R	Population-based cohort study; Netherlands	68.0(NR)	74.0	5.0(NR)	MOF	Medical records	11.0	7578
Model validation^n^										
Ensrud 2009^[60]^	FRAX; 4	P	Study of osteoporotic fractures; US	71.3(5.1)	100	9.2(1.8)	MOF	Self-reported and confirmed by radiographic reports	Hip: 6.2 MOF: 16.6	6252
Hundrup 2010^[61]^	WHI; 1	P	Danish Nurse Cohort Study; Denmark	61.0(6.9)	100	5.0(NR)	Hip fracture	Medical records	0.9	13353
Leslie 2010^[62]^	FRAX; 4	R	Manitoba bone density program; Canada	M: 68.2(10.1) F: 65.7(9.8)	92.7	10.0(NR)	MOF	Medical records	Hip: 1.4 MOF: 6.4	39603
Sornay- Rendu 2010^[63]^	FRAX; 2	P	Os des femmes de Lyon cohort; France	58.8(10.3)	100	10.0(NR)	MOF	Self-reported and confirmed by radiographic reports	MOF: 13.4	867
Trémollieres 2010^[64]^	FRAX; 1	P	Menopause et Os cohort study; US	54.0(4.0)	100	13.4(1.4)	MOF	Self-reported and confirmed by radiographic reports	6.6	2196
Bolland 2011^[65]^	FRAX, Garvan; 6	P	Population-based cohort study; New Zealand	74.2(4.2)	100	8.8(2.4)	Any fractures^j^	Self-reported	Hip: 4.0 FRAX: 16.1 Garvan: 19.6	1422
Langsetmo 2011^[66]^	Garvan; 4	P	Osteoporosis epidemiology study; Canada	M: 67.6(7.6) F: 67.7(7.6)	72.1	M: 8.3(NR) F: 8.6(NR)	MOF	Self-reported	Hip: NR(M/F) MOF: 7.2(M) 14.0(F)	M: 1606 F: 4152
Pressman 2011^[67]^	FRAX; 2	R	Population-based cohort study; US	NR	100	6.6(NR)	Hip fracture	Medical records	1.7	94489
Tanaka 2011^[68]^	FRISC; 1	R	Population-based cohort study; Japan	63.3(10.8)	100	10.0(NR)	MOF	Radiographic reports	18.4	765
Collins 2011^[69]^	QFracture; 4	P	Health improvement network database; UK	M: 47.0(NR) F: 48.0(NR)	50.6	10.0(NR)	MOF	Medical records	MOF: 0.1(M) 0.3(F) Hip: 0.1(M/F)	M: 1108219 F: 1136417
Fraser 2011^[70]^	FRAX; 4	P	Multi-centre osteoporosis study; Canada	M: 65.3(9.1) F: 65.8(8.8)	40.2	10.0(NR)	MOF	Self-reported and confirmed by a doctor	MOF: 6.4(M) 12.0(F) Hip: 2.4(M) 2.7(F)	6697
Azagra 2012^[71]^	FRAX; 4	P	Fracture risk factors and bone densitometry type central dual X-ray cohort; Spain	56.8(8.0)	100	10.0(NR)	MOF	Self-reported and confirmed by medical records	MOF: 8.4 Hip: 2.2	770
Cheung 2012^[72]^	FRAX; 4	P	Mr. and Ms. Os study; China	62.1(8.5)	100	4.5(2.8)	MOF	Self-reported and confirmed by medical records	MOF: 4.7 Hip: 0.9	2266
González- Macías 2012^[73]^	FRAX; 2	P	Ecografía Oseaen Atención Primaria cohort study; Italy	72.3(5.3)	100	3.0(NR)	MOF	Radiographic reports	Hip: 1.0 MOF: 3.8	5201
Briot 2013^[74]^	FRAX; 2	P	Osteoporosis and ultrasound study; Germany	74.2(NR)	100	6.0(NR)	MOF	Self-reported and confirmed by radiographic reports	MOF: 4.9	1748
Czerwiński 2013^[75]^	FRAX; 1	R	Cra cow Medical Centre data; Poland	63.8(6.7)	100	11.0(NR)	MOF	Self-reported	22.1	5092
Cordomí 2013^[76]^	FRAX; 1	R	Centre for technical studies with radioactive isotopes; Spain	56.8(7.8)	100	11.0(NR)	MOF	Self-reported	18.1	1231
Ettinger 2013^[77]^	FRAX; 4	R	Osteoporotic fractures in men study; US	73.5(5.8)	0	8.4(2.3)	MOF	Medical records	Hip: 2.7 MOF: 6.4	5891
Rubin 2013^[78]^	FRAX; 1	P	Population-based cohort study; Denmark	64.0(13.0)	100	3.0(NR)	MOF	Medical records	4.0	3614
Ahmed 2014^[79]^	Garvan; 4	R	Tromsø study; Australia	NR	54.7	M: 7.1(NR) F: 6.9(NR)	MOF	Medical records	M: 1.2 F: 3.2	2992
Friis- Holmberg 2014^[80]^	FRAX; 4	P	Health examination survey; Denmark	M: 58.3(10.6) F: 56.8(10.2)	59.2	4.3(NR)	MOF	Medical records	Hip: 0.4 MOF: 3.1	12758
Van Geel 2014^[81]^	FRAX, Garvan; 7	P	Ten general practice centers cohort study; Netherlands	67.8(5.8)	100	5.0(NR)	MOF	Self-reported and confirmed by radiographic reports	Hip: 1.2 MOF: 9.5	506
Klop 2016^[82]^	FRAX; 2	R	Clinical practice research Datalink cohort study; UK	62.9(11.4)	67.8	9.0(NR)	MOF	Medical records	Hip: 1.4 MOF: 5.0	38755
Orwoll 2017^[83]^	FRAX; 6	R	Osteoporotic fractures in men study; Sweden, US, China	75.0(3.0)^o^ 74.0(6.0)^p^ 72.0(5.0)^q^	0	10.6(NR)^o^ 8.6(NR)^p^ 9.8(NR)^q^	MOF	Medical records or radiographic reports	Hip: 6.8^o^, 3.2^p^, 3.1^q^ MOF: 16.4^o^, 7.2^p^, 3.1^q^	2542^o^ 1469^p^ 1476^q^
Dagan 2017^[84]^	QFracture, FRAX, Garvan; 6	R	Electronic health record; Israel	NR	54.6	4.7(NR)	MOF	Medical records	MOF: 7.7 Hip: 2.7	1054815
Holloway 2018^[85]^	FRAX; 2	P	Geelong osteoporosis study; Australia	70.0(NR)	0	9.5(NR)	MOF	Radiographic reports	Hip: 2.4 MOF: 8.5	591
Crandall 2019^[86]^	FRAX, Garvan; 4	P	Women’s Health Initiative observational study; US	57.9(4.1)	100	10.0(NR)	MOF	Medical records or self-reported	Hip: 0.7 MOF: 8.4	Hip: 62723 MOF: 63621
Holloway- Kew 2019^[87]^	FRAX, Garvan; 8	P	Geelong osteoporosis Study; Australia	M: 69.0(NR) F: 71.0(NR)	49.6	10.0(NR)	MOF	Radiographic reports	M: 8.9 F: 14.2	M: 821 F: 809
Su 2019(2)^[88]^	FRAX+TBS, FRAX; 4	P	Mr. and Ms. Os study; China	M: 72.3(4.9) F: 72.5(5.3)	50.3	M: 9.9(2.8) F: 8.8(1.5)	MOF	Medical records or self-reported	M: 6.6 F: 11.0	M: 1923 F: 1950
Tamaki 2019^[89]^	FRAX+TBS, FRAX; 4	P	Population-based cohort study; Japan	58.1(10.6)	100	10.0(NR)	MOF	Radiographic reports	4.3	1541

F: female; FRA-HS: fracture health search; FRAMO: fracture and mortality index; FRAX: fracture risk assessment tool; FREM: fracture risk evaluation model; FRISC: fracture and immobilization score; FRISK: fracture risk; Garvan: Garvan Fracture Risk Calculator; gSOS: genomic speed of sound; KFRS: Korean fracture risk score; M: male; NR: not reported; MOF: major osteoporotic fracture; MST: mandibular sparse trabeculation; P: prospective cohort study; R: retrospective cohort study; S: sarcopenia; TBS: trabecular bone score; WHI: women's health initiative;

a Naming of models or tools, and No. refers to the number of models that were developed or the number of times models was externally validated in the article.

b Development of new model;

c Included hip, vertebrae (symptomatic), wrist, meta-carpal, humerus, scapula, clavicle, distal femur, proximal tibia, patella, pelvis and sternum;

d Included the Rotterdam Study, The European Vertebral Osteoporosis Study (later the European Prospective Osteoporosis Study), The Canadian Multicentre Oosteoporosis Study (CaMos), Rochester, Sheffield, Dubbo, a cohort from Hiroshima and two cohorts from Gothenburg;

e Included hip, wrist, vertebral, forearm or humerus fractures;

f Included hip fracture, surgical neck fracture of the humerus, distal forearm fracture, or clinical vertebral fracture;

g Included Australia, Belgium, Canada, France, Germany, Italy, The Netherlands, Spain, the United Kingdom, and the United States;

h Included ankle, clavicle, elbow, face, foot, finger, hand, heel, hip, humerus, knee, lower leg, pelvis, rib, toe, upper leg, or wrist fractures;

i Included the UK Biobank, the United States-based Osteoporotic Fractures in Men Study, the Sweden-based Osteoporotic Fractures in Men Study, the Study of Osteoporotic Fractures, and the China Kadoorie Biobank;

j FRAX-defined osteoporotic fractures were fractures of the shoulder, hip, or forearm and clinical vertebral fractures; Garvan-defined osteoporotic fractures were fractures of the hip, vertebrae (symptomatic), forearm, metacarpal, humerus, scapula, clavicle, distal femur, proximal tibia, patella, pelvis, or sternum

k The study not only developed new models, but also externally verified the existing models.

l The study developed and externally verified new models.

m The study not only developed and externally verified new models, but also externally verified the existing models.

n External validation of existing model;

o Sweden.

p US.

q China.

#### Sample size

The sample size of included models ranged from 405 to 12,011,134, and the incidence of fracture ranged from 0.1% to 31.4%. The EPV ranged from 0.1 to 6,613.3. Of the 70 models, 30 (43%) had an EPV less than 20, indicating the existence of over-model fitting (Table 1 andTable 2).

#### Predictors

The number of predictors included in development models ranged from 2 to 21,717 (2 models did not report related information). Most models contained less than 15 predictors (n=55, 79%), while three (4%) models included more than 100 predictors (Table 2). Most models (n=31, 44%) contained some similar predictors, including age, prior fractures, and body mass index (BMI). Other commonly selected predictors were smoking status (n=35, 50%), BMD (n=31, 44%), alcohol use (n=30, 43%), rheumatoid arthritis (n=28, 40%). Sex was included in 25 (36%) models. However, most models were sex-specific, with 23 (33%) models for males only while 31 (44%) for females only. All three models with more than 100 predictors included single nucleotide polymorphisms (SNPs) as predictors (Supplementary table 2).

#### Modelling

Most prediction models (n=42, 60%) were developed using Cox proportional hazards regression, followed with Logistic regression (n=12, 17%), machine learning (n=7, 10%), and Poisson regression (n=7, 10%), while the remaining two (3%) did not report related information.

#### Performance

Sixty-nine (99%) models reported information about discrimination, with AUC or C index ranging from 0.60 to 0.91. To be specific, two (3%) models showed outstanding discrimination, nine (13%) showed excellent discrimination, 39 (57%) showed acceptable discrimination, and 20 (57%) showed poor discrimination. Calibration was reported among 25 (36%) models, with all of them being judged as good fitness. Calibration was assessed using Hosmer-Lemeshow test (n=11, 16%), the calibration slope (n=7, 10%), and the calibration intercept (n=7, 10%). Thirty-three (47%) models were internally validated using training test split (n=17), bootstrapping (n=9), and cross validation (n=7). It is worth noting that only four (6%) models used suitable methods for both internal validation (using bootstrapping or cross validation) and calibration calculation (using calibration slope or calibration intercept) (Table 2).

**Table 2:** Information related to predictive model of included studies.

Author	Type of predictive model	EPV	No. of included predictors	Modeling method	Type of validation	Performance^a^ (95% CI, if reported)			
						AUC/C index	Sensitivity	Specificity	Calibration
**WHI (women's health initiative)**									
Robbins 2007^[29]^	Development and internal validation^b^	27.3	10	Cox’s proportional hazards	Cross validation	0.80(0.77 to 0.82)	NR	NR	*P*=0.20^h^
Hundrup 2010^[61]^	External validation^c^	12.2	10	Logistic regression	Geographical validation	0.82	0.69	0.80	1.08^i^
**FRAMO (fracture and mortality index)**									
Albertsson 2007^[28]^	Development only	1.4	4	Cox’s proportional hazards	NA	0.72(0.64 to 0.81)	0.81	0.64	NR
**Garvan (Garvan Fracture Risk Calculator)**									
Nguyen 2008^[12]^	Development and internal validation	M: 11.5 F: 32.8	4	Cox’s proportional hazards	Bootstrapping	Model 1: 0.75(M/F) Model 2: 0.74(M), 0.72(F)	NR	NR	0.01 to 0.02^j^
Bolland 2011^[65]^	External validation	Hip: 11.4 MOF: 55.8	5	NR	Geographical validation	Hip: 0.67 (0.60-0.75)^k^ MOF: 0.64 (0.60-0.67)^k^	NR	NR	*P*<0.01^h^
Henry 2011^[34]^	External validation	25.0	5	NR	Geographical validation	0.70(0.65 to 0.75)	NR	NR	NR
Langsetmo 2011^[66]^	External validation	Hip: NR(M/F) MOF: 29.0(M) 145.8(F)	4	Cox’s proportional hazards	Geographical validation	Hip: 0.85(M), 0.80(F) MOF: 0.69(M), 0.70(F)	NR	NR	NR
Van Geel 2014^[81]^	External validation	Hip: 1.5 MOF: 12.0	5	NR	Geographical validation	Model 1: 0.70(hip), 0.70(MOF) Model 2: NR(hip), 0.65(MOF)	NR	NR	NR
Ahmed 2014^[79]^	External validation	71.2	5	NR	Geographical validation	Model 1: 0.61(M), 0.62(F) Model 2: 0.57(M), 0.58(F)	NR	NR	NR
Dagan 2017^[84]^	External validation	Hip: 5618.2 MOF: 16312.8	5	NR	Geographical validation	Hip: 0.78^k^ MOF: NR^k^	Hip: 0.57 MOF: NR	Hip: 0.81 MOF: NR	0.68^i^
Crandall 2019^[86]^	External validation	Hip: 87.8 MOF: 1068.8	4	Logistic regression	Geographical validation	Hip: 0.57(0.55 to 0.60) MOF: 0.57(0.57 to 0.58)	Hip: 0.81 MOF: 0.16	Hip: 0.31 MOF: 0.94	NR
Holloway- Kew 2019^[87]^	External validation	M: 3.4 F: 8.4	5	Logistic regression	Geographical validation	Model 1: 0.68(0.63 to 0.73)(M) 0.70(0.65 to 0.74)(F) Model 2: 0.67(0.62 to 0.72)(M) 0.67(0.62 to 0.71)(F)	NR	NR	NR
**FRAX (fracture risk assessment tool)**									
Kanis 2008^[10]^	Development and external validation^d^	Hip: 77.3 MOF: 301.6	11	Poisson regression	Geographical validation	Hip: 0.66^n^, 0.74^o^ MOF: 0.60^n^, 0.62^o^	NR	NR	NR
Ensrud 2009^[60]^	External validation	Hip: 35.4 MOF: 94.3	11	Logistic regression	Geographical validation	Hip: 0.71^n^, 0.75^o^ MOF: 0.61^n^, 0.68^o^	NR	NR	NR
Leslie 2010^[62]^	External validation	Hip: 49.9 MOF: 231.2	11	Cox’s proportional hazards	Geographical validation	Hip: 0.79(0.78 to 0.81)^n^ 0.83(0.82 to 0.85)^o^ MOF: 0.66(0.65 to 0.67)^n^ 0.69(0.68 to 0.71)^o^	NR	NR	Hip: 0.92(M) 1.03(F)^i^ MOF: 1.24)(M) 1.13(F)^i^
Sornay- Rendu 2010^[63]^	External validation	MOF: 1.5	11	NR	Geographical validation	0.75(0.71 to 0.79)^n^ 0.78(0.72 to 0.82)^o^	NR	NR	NR
Trémollieres 2010^[64]^	External validation	13.2	11	Cox’s proportional hazards	Geographical validation	0.63(0.56 to 0.69)^o^	NR	NR	NR
Yun 2010^[31]^	External validation	Hip: 17.0 MOF: 39.1	11	Logistic regression	Geographical validation	Hip: 0.64(0.60 to 0.68)^o^ MOF: 0.55(0.53 to 0.58)^o^	NR	NR	NR
Bolland 2011^[65]^	External validation	Hip: 5.2 MOF: 20.8	11	NR	Geographical validation	Hip: 0.69 (0.63 to 0.76)^n^, 0.70 (0.64 to 0.77)^o^, MOF: 0.62 (0.58 to 0.66)^n^ 0.64 (0.60 to 0.68)^o^	NR	NR	Hip: *P*=0.18^h^,^n^ *P*<0.01^h^,^o^ MOF: *P*<0.01^h^
Pressman 2011^[67]^	External validation	143.5	11	Logistic regression	Geographical validation	0.83(0.82 to 0.84)^n^ 0.84(0.83 to 0.85)^o^	NR	NR	NR
Henry 2011^[34]^	External validation	11.4	11	NR	Geographical validation	0.66(0.61 to 0.71)^n^ 0.68(0.63 to 0.73)^o^	NR	NR	NR
Tamaki 2011^[35]^	External validation	Hip: 3.9 MOF: 0.4	11	Logistic regression	Geographical validation	Hip: 0.86(0.68 to 1.00)^n^ 0.88(0.73 to 1.00)^o^ MOF: 0.67(0.59 to 0.75)^n^ 0.69(0.61 to 0.76)^o^	NR	NR	NR
Fraser 2011^[70]^	External validation	Hip: 15.9 MOF: 63.2	11	Cox’s proportional hazards	Geographical validation	Hip: 0.77(0.73 to 0.80)^n^ 0.80(0.77 to 0.83)^o^ MOF: 0.66(0.63 to 0.68)^n^ 0.69(0.67 to 0.7)^o^	NR	NR	Hip: 1.83(M) 0.93(F)^i^ MOF: 1.26(M) 1.07(F)^i^
Azagra 2012^[71]^	External validation	Hip: 1.5 MOF: 5.9	11	NR	Geographical validation	Hip: 0.89^n^, 0.85^o^ MOF: 0.69^n^, 0.72^o^	NR	NR	*P*>0.05^h^
Cheung 2012^[72]^	External validation	Hip: 1.9 MOF: 9.6	11	Cox’s proportional hazards	Geographical validation	Hip: 0.90(0.83 to 0.97)^n^ 0.88(0.82 to 0.94)^o^ MOF: 0.71(0.66 to 0.76)^n^ 0.73(0.68 to 0.80)^o^	NR	NR	NR
González- Macías 2012^[73]^	External validation	Hip: 5.0 MOF: 18.3	11	NR	Geographical validation	Hip: 0.64^o^ MOF: 0.62^o^	NR	NR	NR
Briot 2013^[74]^	External validation	7.7	11	Logistic regression	Geographical validation	0.62(0.56 to 0.68)^n^ 0.66(0.60 to 0.73)^o^	NR	NR	NR
Czerwiński 2013^[75]^	External validation	29.5	11	NR	Geographical validation	0.59(0.54 to 0.64)^o^	NR	NR	NR
Cordomí 2013^[76]^	External validation	MOF: 20.2	11	NR	Geographical validation	0.61(0.57 to 0.65)^o^	NR	NR	NR
Ettinger 2013^[77]^	External validation	Hip: 14.6 MOF: 34.0	11	Logistic regression	Geographical validation	Hip: 0.69^n^, 0.77^o^ MOF: 0.63^n^, 0.67^o^	NR	NR	NR
Rubin 2013^[78]^	External validation	15.6	10^o^	Cox’s proportional hazards	Geographical validation	0.72(0.69, 0.76)^n^	NR	NR	NR
Friis- Holmberg 2014^[80]^	External validation	Hip: 4.9 MOF: 35.9	11	Cox’s proportional hazards	Geographical validation	MOF: 0.67(0.61 to 0.73)^o^(M) 0.72(0.69 to 0.75)^o^(F) Hip: 0.72(0.60 to 0.84)^o^(M) 0.86(0.81 to 0.92)^o^(F)	NR	NR	NR
Van Geel 2014^[81]^	External validation	Hip: 0.5 MOF: 4.4	11	NR	Geographical validation	Hip: 0.70^o^ MOF: 0.65^n^, 0.69^o^	NR	NR	NR
Yu 2014^[39]^	External validation	Hip: 12.0 MOF: 51.3	11	Cox’s proportional hazards	Geographical validation	Hip: 0.70^n^(M), 0.76^o^(M) 0.73^n^(F), 0.76^o^(F) MOF: 0.61^n^(M), 0.64^o^(M) 0.60^n^(F), 0.62^o^(F)	NR	NR	NR
Iki 2015^[40]^	External validation	2.8	11	Logistic regression	Geographical validation	0.68(0.59 to 0.78)^o^	NR	NR	NR
Klop 2016^[82]^	External validation	Hip: 48.7 MOF: 175.0	10^o^	Logistic regression	Geographical validation	Hip: 0.83^n^ MOF: 0.71^n^	NR	NR	1.02^i^
Orwoll 2017^[83]^	External validation	NR	11	Logistic regression	Geographical validation	Hip: 0.72^p^, 0.78^q^, 0.74^r^ MOF: 0.65^p^, 0.65^q^, 0.69^r^	NR	NR	NR
Sundh 2017^[48]^	External validation	7.1	10^o^	NR	Geographical validation	0.75(0.70 to 0.81)^n^	NR	NR	NR
Dagan 2017^[84]^	External validation	Hip: 2553.7 MOF: 7414.9	11	NR	Geographical validation	Hip: 0.82^o^ MOF: 0.71^o^	Hip: 0.66 MOF: 0.47	Hip: 0.81 MOF: 0.82	0.94^i^
Biver 2018^[50]^	External validation	12.8	11	Cox’s proportional hazards	Geographical validation	0.71^o^	NR	NR	NR
Su 2018^[52]^	External validation	M: 12.6 F: 21.5	11	Cox’s proportional hazards	Geographical validation	M: 0.69(0.64 to 0.73)^o^ F: 0.61(0.58 to 0.65)^o^	NR	NR	NR
Holloway 2018^[85]^	External validation	Hip: 1.3 MOF: 4.5	11	NR	Geographical validation	Hip: 0.74^o^ MOF: 0.85^o^	Hip: 0.57 MOF: 0.02	Hip: 0.84 MOF: 0.99	NR
Crandall 2019^[86]^	External validation	Hip: 39.9 MOF: 485.8	10^o^	Logistic regression	Geographical validation	Hip: 0.64(0.61 to 0.66)^n^ MOF: 0.58(0.57 to 0.59)^n^	Hip: 0.81 MOF: 0.59	Hip: 0.81 MOF: 0.68	NR
Holloway- Kew 2019^[87]^	External validation	M: 7.3 F: 11.5	10^o^	Logistic regression	Geographical validation	M: 0.70(0.65 to 0.76)^n^ 0.72(0.67 to 0.78)^o^ F: 0.74(0.69 to 0.78)^n^ 0.75(0.71 to 0.80)^o^	NR	NR	NR
Su 2019(1)^[53]^	External validation	17.3	10^o^	Cox proportional hazard	Geographical validation	0.70(0.67 to 0.74)^n^	0.62	0.78	NR
Su 2019(2)^[88]^	External validation	M: 11.5 F: 19.5	11	Cox proportional hazard	Geographical validation	M: 0.68(0.63 to 0.73)^o^ F: 0.63(0.59 to 0.67)^o^	NR	NR	NR
Tamaki 2019^[89]^	External validation	6.1	11	Logistic regression	Geographical validation	0.67(0.61 to 0.73)^n^ 0.68(0.62 to 0.74)^o^	NR	NR	NR
Lu 2021^[58]^	External validation	Hip: 776.0 MOF: 1862.4	11	Cox’s proportional hazards	Geographical validation	MOF: 0.76(0.75 to 0.76)^o^ Hip: 0.81(0.80 to 0.81)^o^	NR	NR	NR
**FRAX+S (fracture risk assessment tool and sarcopenia)**									
Yu 2014^[39]^	Development only^e^	Hip: 11.0 MOF: 47.1	12	Cox’s proportional hazards	NA	Hip: 0.73^n^, 0.78^o^(M) 0.73^n^, 0.75^o^(F) MOF: 0.62^n^, 0.66^o^(M) 0.60^n^, 0.62^o^(F)	NR	NR	NR
**FRAX+TBS (fracture risk assessment tool and trabecular bone score)**									
Iki 2015^[40]^	Development only	0.1	12	Logistic regression	NA	0.68(0.57 to 0.80)^o^	NR	NR	NR
Su 2019(2)^[88]^	External validation	M: 10.6 F: 17.8	12	Cox proportional hazard	Geographical validation	M: 0.69(0.65 to 0.74)^o^ F: 0.63(0.59 to 0.67)^o^	NR	NR	NR
Tamaki 2019^[89]^	External validation	5.6	12	Logistic regression	Geographical validation	0.68(0.62 to 0.74)^n^ 0.68(0.62 to 0.74)^o^	NR	NR	NR
**FRAX+MST (fracture risk assessment tool and mandibular sparse trabeculation)**									
Sundh 2017^[48]^	Development only	5.9	11^o^	NR	NA	0.75(0.70 to 0.81)^n^	NR	NR	NR
**FRAX+FALL (fracture risk assessment tool and history of falls)**									
Su 2018^[52]^	Development only	M: 11.6 F: 19.7	12	Cox’s proportional hazards	NA	M: 0.69(0.65 to 0.74)^o^ F: 0.61(0.58 to 0.65)^o^	NR	NR	NR
**QFracture**									
Hippisley- Cox 2009^[11]^	Development and internal validation	Hip: 161.4(M) 489.6(F) MOF: 417.6(M) 1281.6(F)	M: 12 F: 17	Cox’s proportional hazards	Training test split	Hip: 0.86(0.85 to 0.86)(M) 0.89(0.89 to 0.89)(F) MOF: 0.69(0.68 to 0.69)(M) 0.79(0.79 to 0.79)(F)	NR	NR	0.99^i^
Collins 2011^[69]^	External validation	Hip: 274.8(M) 833.2(F) MOF: 559.4(M) 1732.3(F)	M: 12 F: 17	NR	Geographical validation	Hip: 0.86(M), 0.89(F) MOF: 0.74(M), 0.82(F)	NR	NR	Hip: 0.01(M) 0.01(F)^j^ MOF: 0.01(M) 0.03(F)^j^
**Updated QFracture**									
Hippisley- Cox 2012^[36]^	Development and internal validation	Hip: 166.6(M) 479.5(F) MOF: 461.2(M) 1467.0(F)	M: 26 F: 25	Cox’s proportional hazards	Training test split	Hip: 0.88(0.87 to 0.88 )(M) 0.89(0.89 to 0.90) (F) MOF: 0.71(0.70 to 0.72) (M) 0.79(0.79 to 0.79) (F)	Hip: 0.64(M) 0.60(F) MOF: 0.37(M) 0.35(F)	NR	*P*>0.05^h^
Dagan 2017^[84]^	External validation	Hip: 906.2 MOF: 2631.1	31	NR	Geographical validation	Hip: 0.88 MOF: 0.71	Hip: 0.70 MOF: 0.46	Hip: 0.81 MOF: 0.82	0.60^i^
**FRISC (fracture and immobilization score)**									
Tanaka 2010^[30]^	Development and external validation	23.9	5	Poisson regression	Geographical validation	0.73(0.66 to 0.79)	NR	NR	*P*=0.17^h^
Tanaka 2011^[68]^	External validation	28.2	5	Cox’s proportional hazards	Geographical validation	0.73(0.69 to 0.78)	NR	NR	NR
**FRISK (fracture risk)**									
Henry 2011^[34]^	Development only	25.0	5	NR	NA	0.66(0.60 to 0.71)	59.2	0.65	NR
**KFRS (Korean fracture risk score)**									
Kim 2016^[42]^	Development and internal validation	M: 543.2 F: 1661.2	9	Cox’s proportional hazards	Training test split	M: 0.68, F: 0.65	NR	NR	1.00^i^
**FRA-HS (fracture health search)**									
Francesco 2017^[43]^	Development and external validation	6613.3	9	Cox’s proportional hazards	Geographical validation	0.85	NR	NR	1.00(0.83 to 1.18)^i^
**FREM (fracture risk evaluation model)**									
Rubin 2018^[51]^	Development and internal validation	M: 2.3 F: 5.7	M: 44 F: 39	Logistic regression	Training test split	M: 0.75(0.74 to 0.76) F: 0.75(0.74 to 0.80)	NR	NR	0.01^j^
**GSOS (genomic speed of sound)**									
Lu 2021^[58]^	Development, internal and external validation^f^	MOF: <0.1 Hip: <0.1	21717	Cox’s proportional hazards	Training test split, geographical validation	MOF: 0.73(0.73 to 0.74) Hip: 0.80(0.79 to 0.81)	NR	NR	NR
**Models without a specific name**									
Dargent- Molina 2002^[25]^	Development only	NR	5	Cox’s proportional hazards	NA	NR	0.37	0.85	NR
Colón- Emeric 2002^[26]^	Development and external validation	Hip: 11.7 Any: 33.7	Hip: 7 Any: 6	Logistic regression	Geographical validation^g^	Hip: 0.75 Any: 0.57	NR	NR	NR
McGrother 2002^[27]^	Development and internal validation	1.4	6	Cox’s proportional hazards	Cross validation	0.82	0.67(0.54 to 0.80)	0.68(0.65 to 0.72)	NR
Yun 2010^[31]^	Development only	NR	NR	Logistic regression	NA	Hip: 0.74(0.70 to 0.77) MOF: 0.71(0.69 to 0.73)	NR	NR	NR
Sambrook 2011^[32]^	Development only	NR	2	Cox’s proportional hazards	NA	0.78	NR	NR	NR
Bow 2011^[33]^	Development only	1.1	7	Cox’s proportional hazards	NA	0.82	NR	NR	NR
Tamaki 2011^[35]^	Development only	Hip: 0.4 MOF: 3.9	3	Logistic regression	NA	Hip: 0.90(0.77 to 1.00) MOF: 0.71(0.63 to 0.79)	NR	NR	NR
LaFleur 2012^[37]^	Development and internal validation	NR	Hip: 10 MOF: 12	Cox’s proportional hazards	Bootstrapping	Hip: 0.81 MOF: 0.74	0.84	0.75	NR
Schousboe 2014^[38]^	Development and internal validation	172.1	7	Logistic regression	Bootstrapping	0.69	NR	NR	*P*>0.05^h^
Jang 2016^[41]^	Development only	M: 4.0 F: 5.6	M: 5 F: 7	Logistic regression	NA	M: 0.74, F: 0.73	NR	NR	*P*>0.05^h^
Kruse 2017^[44]^	Development and internal validation	M: <0.1 F: 0.2	M: 9 F: 11	Machine learning	Bootstrapping	M: 0.89(0.82 to 0.95) F: 0.91(0.88 to 0.93)	M: 0.69 F: 0.88	M: 0.69 F: 0.81	NR
Li 2017^[45]^	Development only	11.5	5	Cox’s proportional hazards	NA	0.71	NR	NR	NR
Su 2017^[46]^	Development only	M: 21.0 F: 35.8	2	Poisson regression	NA	M: 0.67(0.62 to 0.71) F: 0.58(0.55 to 0.62)	M: 0.64 F: 0.69	M: 0.74 F: 0.42	NR
Weycker 2017^[47]^	Development only	NR	Hip: 5 Non vertebral: 7	Cox’s proportional hazards	NA	Hip: 0.71(0.67 to 0.76) Non vertebral: 0.62(0.59 to 0.65)	NR	NR	*P*=0.41^h^
Biver 2018^[50]^	Development only	8.3	12	Cox’s proportional hazards	NA	0.76	NR	NR	NR
Reber 2018^[49]^	Development and internal validation	436.9	3	Cox’s proportional hazards	Training test split	0.70(0.69 to 0.71)	NR	NR	NR
Su 2019(1)^[53]^	Development and internal validation	Model 1: 57.3 Model 2: 13.2	Model 1: 3 Model 2: 13	Machine learning	Cross validation	Model 1: 0.71(0.68 to 0.75) Model 2: 0.73(0.69 to 0.76)	NR	NR	NR
Engels 2020^[54]^	Development and internal validation	80.6	23	Machine learning	Training test split	0.70(0.68 to 0.71)	NR	NR	0.03^j^
Kong 2020^[55]^	Development and internal validation	19.9	21	Machine Learning	Cross validation	0.69	NR	NR	NR
Sheer 2020^[56]^	Development and internal validation	1896.5	6	Cox’s proportional hazards	Training test split	0.71	NR	NR	NR
Wu 2020^[57]^	Development and internal validation	0.4	1115	Machine learning	Cross validation	0.71	NR	NR	NR
de Vries 2021^[59]^	Development and internal validation	18.3	8	Cox’s proportional hazards	Cross validation	0.70(0.66 to 0.73)	NR	NR	NR

AUC: area under receiver operating characteristic curve; EPV: events per variable; M: male; MOF: major osteoporotic fracture; NA: not applicable; NR: not reported;

a Performance is given for the strongest form of validation reported;

b Development and internal validation refers to the study developed and internally validated the new model;

c External validation refers to the study only externally validated the existing model;

d Development and external validation refers to the study developed and externally validated the new model;

e Development only refers to the study only developed the new model;

f Development, internal and external validation refers to the study developed, internally and externally validated the new model;

g External validation in different population only;

h *P*value refers to the results of Hosmer-Lemeshow test;

i Refers to value of calibration slope;

j Refers to value of calibration intercept;

k The type of model used is not reported;

n Without bone mineral density;

o With bone mineral density;

p Sweden;

q US;

r China;

#### Model presentation

Only 39 (56%) models provided model presentation as a web calculator, nomogram, or risk score of each predictor to allow practical use, while the remaining 31 (44%) models did not offer related information.

### Risk of bias and applicability

All 70 models were judged as high overall risk of bias. Respectively 31 (44%) and 10 (14%) models had an unclear and high risk of bias in the outcome domain. Mainly because it is unclear whether a prespecified or standard outcome definition or subjective outcome measures (e.g., self-reported) had been used. All models (n=70, 100%) were at high risk of bias for the analysis domain, which is commonly due to the risk of overfitting caused by an insufficient number of cases, or categorization of continuous predictors. In addition, the calibration of many models was not assessed or was not assessed correctly (e.g., using Hosmer-Lemeshow test). In terms of applicability, 44 (63%) models had a low concern while the remaining 26 (37%) had a high concern. The most common concern about applicability was the outcome domain, which focused on hip fracture. The models focused on predicting hip fracture may not accurately predict all osteoporosis fractures. Details on the risk of bias and applicability assessments are presented inFigure 2 and Supplementary table 3.

### Studies focus on external validation of OF prediction model

In 44 articles [10,26,30,31,34,35,39,40,43,48,50,52,53,58,60-89], 138 external validations were performed. However, most (n=48, 69%) of the 70 developed models has never been externally validated. Out of the 22 (31%) models externally validated, 15 (21%) were validated once, and five (7%) were validated more than five times (range: 5 to 37). The most commonly validated models were FRAX with BMD (for MOF) (n=37, 27%) and FRAX with BMD (for hip fracture) (n=23, 17%) (Table 1 andTable 2).

**Figure 2.**
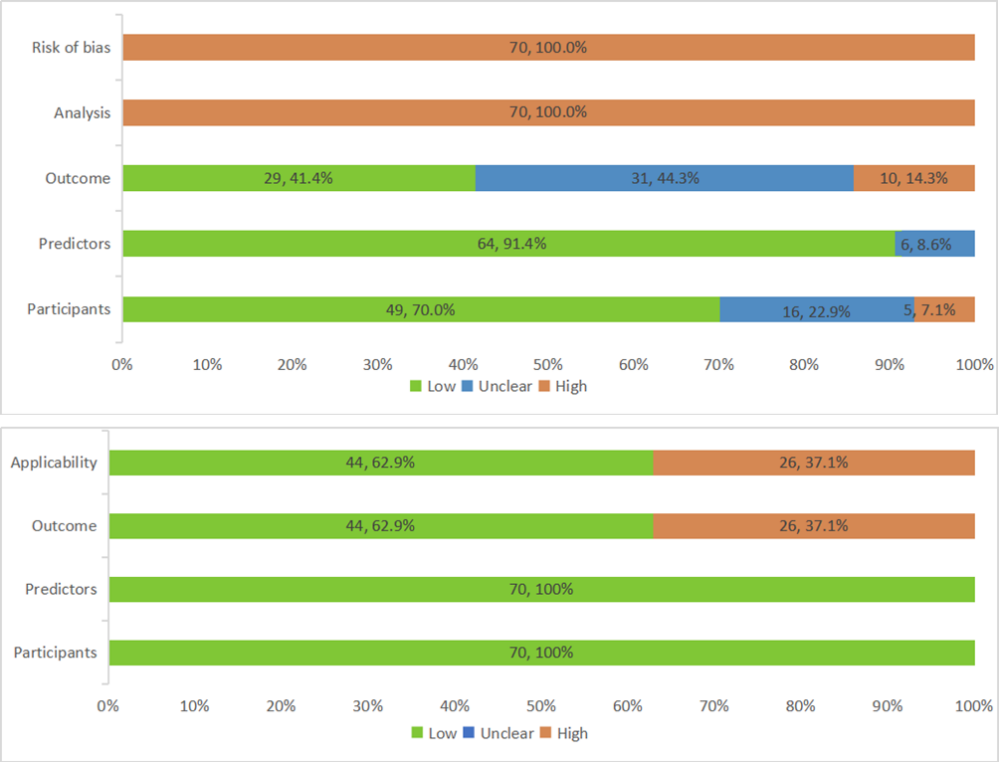
Summary results on risk of bias and applicability assessment (using PROBAST) of development of osteoporotic fracture prediction models.

#### Study populations and outcomes

All the external validations were conducted in a different geographical area from the development study. Most of the participants were from China (n=29, 21%), US (n=27, 20%) or UK (n=16, 12%), with the remaining (n=66, 48%) from countries in Oceania, Western Europe or East Asia. It is worth noting that no external validation was conducted among participants from Africa, South America, and the Middle East. Most models (n=109, 79%) were sex-specific, with 76 (55%) being validated for female, and 33 (24%) for male. The average age of participants ranged from 54 to 75 years. The outcomes included MOF (n=84, 61%), hip fracture (n=50, 36%) and any fractures (n=4, 9%). Diagnosis of fracture was mostly through medical records (n=57, 41%), following with self-reported (n=28, 20%), self-reported with another confirmation method (n=28, 20%) and radiograph reports (n=25, 18%) (Table 1).

#### Sample size

The sample size ranged from 412 to 1,136,417, and the incidence of fracture ranged from 0.1% to 22.1%. The EPV ranged from 0.1 to 16,312.8, and 114 (83%) models were less than 100, indicating the existence of over model fitting (Table 1 andTable 2).

#### Performance

The discrimination of 136 (99%) models was reported as an AUC or C index (range: 0.55 to 0.90). Among them, one (1%) showed outstanding discrimination, 23 (15%) showed excellent discrimination, 45 (38%) showed acceptable discrimination, and 67 (38%) showed poor discrimination. Calibration measurements were reported for 33 (24%) models, with 31 (22%) models showing good fitness. Calibration was assessed with calibration slope (n=18, 13%), the Hosmer-Lemeshow test (n=11, 8%), and the calibration intercept (n=4, 3%). Only 22 (16%) models used suitable methods (calibration slope or calibration intercept) for calibration calculation (Table 2).

**Figure 3.**
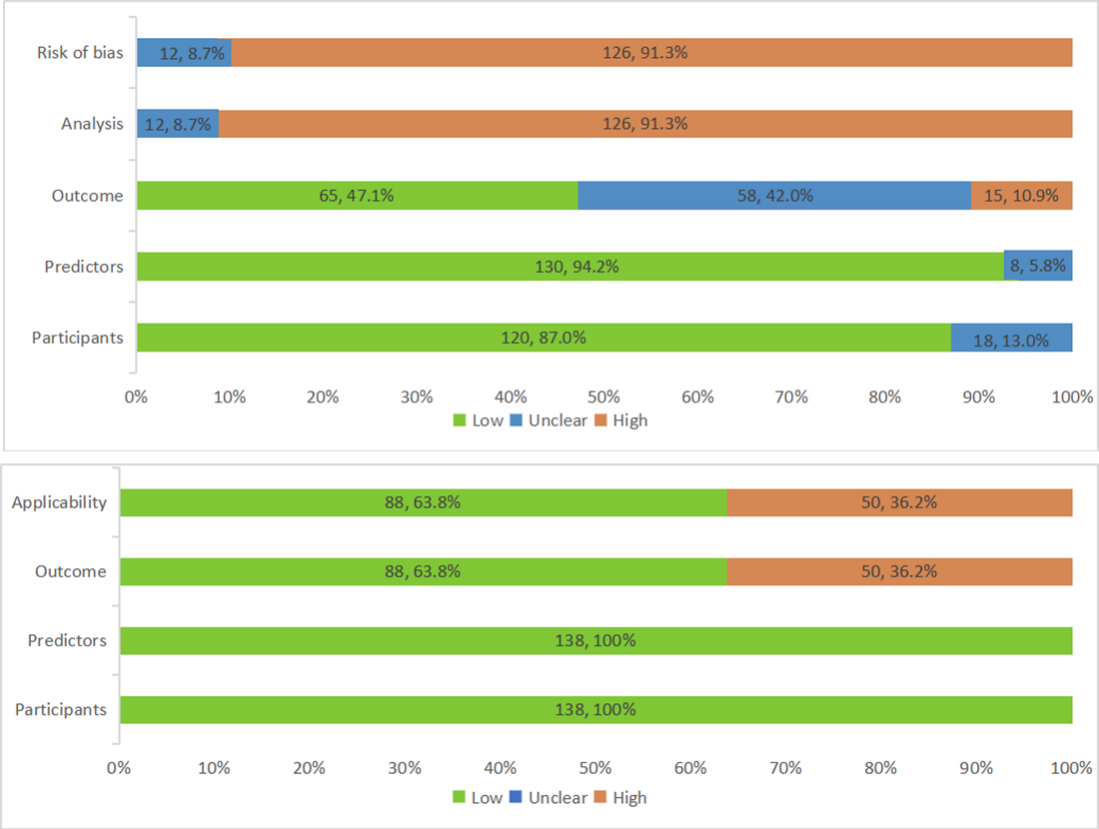
Summary results on risk of bias and applicability assessment (using PROBAST) of external validation of osteoporotic fracture prediction model.

The discrimination of the four most frequently validated models, including FRAX with BMD (for MOF), FRAX with BMD (for hip fracture), FRAX without BMD (for MOF), and FRAX without BMD (for hip fracture), varied among the studies, with AUC/C index ranged from 0.55 to 0.85, 0.64 to 0.88, 0.58 to 0.75, 0.64 to 0.90, respectively. Other commonly validated models, including the Garvan Model 1 and Garvan Model 2 in females, showed AUC/C index between 0.57 to 0.80, 0.58 to 0.78, respectively.

There were some FRAX extension models based on FRAX predictors and other predictors, such as FRAX plus sarcopenia [39], FRAX plus history of falls [52], FRAX plus trabecular bone score (TBS) [40,88,89]. The model performance of the extension models (AUC/C index: 0.60 to 0.78) was slightly improved compared with FRAX alone (AUC/C index: 0.60 to 0.74). However, most of them had not been externally validated yet.

The AUC/C indexes of the models using the machine learning modelling method were between 0.69 and 0.91, indicating relatively good discrimination. Some models only included two or three predictors, such as Sambrook 2011 (age, prior fractures) [32], Su 2017 (TBS, femoral neck BMD) [46], Tamaki 2011 (age, weight, femoral neck BMD) [35], with AUC/C indexes being 0.78, 0.67, and 0.90, respectively. Wu 2020 [57], gSOS (for MOF) [58], and gSOS (for hip fracture) [58] included SNPs as predictors, all contained more than 1000 predictors, with AUC/C indexes being 0.71, 0.73 and 0.80, respectively.

#### Risk of bias and applicability

Most models (n=126, 91%) were judged as high overall risk of bias, while the remaining 12 (9%) were unclear risk of bias, and no low risk of bias model was identified. The most common issues were seen in the analysis domain, in which 126 (91%) models were rated as high risk of bias. The most common reason was the insufficient number of cases or the incorrect assessment of calibration. Several models have an unclear risk (n=58, 42%) or high risk (n=15, 11%) of bias in outcome domain. It is mainly because of the unclarity of whether a prespecified or standard outcome definition or subjective outcome measures (e.g., self-reported) had been used. In applicability section, 88 (64%) models had a low concern, and the remaining 50 (36%) models had a high concern, because they focused on hip fracture in the outcome domain. Details on risk of bias and applicability assessments are presented inFigure 3 and Supplementary table 4.

### Model comparison

FRAX, QFracture, and Garvan were the three most used tools in clinical practice. In addition, there were also some tools with a potential clinical value that had been externally verified with good performance (e.g., FRA-HS, WHI). The details of these models that have been externally validated as well as their advantages and disadvantages were summarized inTable 3.

**Table 3:** Predictors, advantages and disadvantages of externally validated models.

Author	Model	Details of the predictors included in the model	Advantages	Disadvantages
Colón- Emeric 2002^[26]^	Colón-Emeric- Any	Gender, ethnicity, BMI, activity of daily living difficulty, antiepileptic use, Rosow-Breslau impairment^a^	• Relatively easy to measure • Contains few predictors	• Performance is poor • Rarely externally verified • Dose-response is not included
	Colón-Emeric- Hip	Age, gender, ethnicity, BMI, stroke history, cognitive impairment, Rosow-Breslau impairment^a^	• Relatively easy to measure • Contains few predictors	• Performance is acceptable • Rarely externally verified • Dose-response is not included
Robbins 2007^[29]^	WHI	Age, general health, BMI, prior fractures, ethnicity, physical activity, smoking status, family history of fractures, corticosteroid use, treated diabetes	• Easy to measure • Performance is excellent • Includes dose-response for general health and physical activity	• Rarely externally verified • Not applicable to male
Nguyen 2008^[12]^	Garvan-Model 1	Age, femoral neck BMD, prior fractures, history of falls	• Contains few predictors • Includes dose-response for number of prior fractures and falls • Commonly used in clinical practice	• Performances range from poor to acceptable • Need to measure BMD
	Garvan-Model 2	Age, weight, prior fractures, history of falls	• Easy to measure • Contains few predictors • Includes dose-response for number of prior fractures and falls • Commonly used in clinical practice	• Performances range from poor to acceptable
Kanis 2008^[10]^	FRAX-with BMD	Age, gender, BMI, prior fractures, family history of fractures, glucocorticoid use, smoking status, alcohol use, RA, secondary osteoporosis, femoral neck BMD	• Had been externally verified many times • Widely used in clinical practice	• Performances range from poor to acceptable • Need to measure BMD • Dose-response is not included
	FRAX-without BMD	Age, gender, BMI, prior fractures, family history of fractures, glucocorticoid use, smoking status, alcohol use, RA, secondary osteoporosis	• Had been externally verified many times • Widely used in clinical practice • Relatively easy to measure	• Performances range from poor to acceptable. • Dose-response is not included
Hippisley- Cox 2009^[11]^	QFracture-M	Age, BMI, smoking status, alcohol use, RA, cardiovascular disease, type 2 diabetes, asthma, tricyclic antidepressants use, corticosteroids use, history of falls, liver disease	• Performances range from acceptable to excellent • Includes dose-response for smoking, alcohol use, type of diabetes • Commonly used in clinical practice • Relatively easy to measure	• Contains many predictors
	QFracture-F	Hormone replacement therapy use, age, BMI, smoking status, alcohol use, parental history of osteoporosis, RA, cardiovascular disease, type 2 diabetes, asthma, tricyclic antidepressants, corticosteroids use, history of falls, menopausal symptoms, chronic liver disease, gastrointestinal malabsorption, other endocrine disorders	• Performance is excellent • Includes dose-response for smoking, alcohol use, type of diabetes • Commonly used in clinical practice • Relatively easy to measure	• Contains many predictors
Tanaka 2010^[30]^	FRISC	Age, weight, prior fractures, back pain, lumbar BMD	• Contains few predictors	• Performance is acceptable • Need to measure BMD • Not applicable to male • Dose-response is not included
Hippisley- Cox 2012^[36]^	Updated QFracture-F	Age, BMI, ethnicity, alcohol use, smoking status, chronic obstructive pulmonary disease or asthma, any cancer, cardiovascular disease, dementia, epilepsy, history of falls, chronic liver disease, Parkinson’s disease, RA or systemic lupus erythematosus, chronic renal disease, type 1 diabetes, type 2 diabetes, prior fractures, endocrine disorders, gastrointestinal malabsorption, antidepressants, corticosteroids use, unopposed hormone replacement therapy, parental history of osteoporosis	• Performances range from acceptable to excellent • Includes dose-response for smoking, alcohol use, type of diabetes • Commonly used in clinical practice • Relatively easy to measure	• Contains many predictors
	Updated QFracture-M	Age, BMI, ethnicity, alcohol use, smoking status, chronic obstructive pulmonary disease or asthma, any cancer, cardiovascular disease, dementia, epilepsy, history of falls, chronic liver disease, Parkinson’s disease, RA or systemic lupus erythematosus, chronic renal disease, type 1 diabetes, type 2 diabetes, prior fractures, endocrine disorders, gastrointestinal malabsorption, antidepressants, corticosteroids use, unopposed hormone replacement therapy, parental history of osteoporosis, care home residence	• Performances range from acceptable to excellent • Includes dose-response for smoking, alcohol use, type of diabetes • Commonly used in clinical practice • Relatively easy to measure	• Contains many predictors
Iki 2015^[40]^	FRAX+TBS	Age, gender, BMI, prior fractures, family history of fractures, glucocorticoid use, smoking status, alcohol use, RA, secondary osteoporosis, femoral neck BMD, trabecular bone score	• It is an extended model of FRAX-with BMD, with its performance better than that of FRAX-with BMD	• Need to measure BMD • Rarely externally verified • Dose-response is not included
Francesco 2017^[43]^	FRA-HS	Age, gender, prior fractures, secondary osteoporosis, corticosteroids use, RA, BMI, smoking status, alcohol abuse disorder	• Relatively easy to measure • Performance is excellent	• Rarely externally verified • Dose-response is not included
Lu 2021^[58]^	GSOS	21,717 SNP	• Performances range from acceptable to excellent	• Contains many predictors • Predictors are difficult to measure

BMD: bone mineral density; BMI: body mass index; F: female; FRA-HS: Fracture health search; FRAX: fracture risk assessment tool; FRISC: fracture and immobilization score; GSOS: Genomic speed of sound; M: male; RA: rheumatoid arthritis; SNP: Single Nucleotide Polymorphisms; TBS: trabecular bone score; WHI: women's health initiative;

a Rosow-Breslau impairment is defined as difficulty doing heavy work, walking upstairs, or unable to walk a mile.

## DISCUSSION

This systematic review summarized and critically appraised 68 studies focused on OF risk prediction models in the general population, with 70 developed models and 138 external validations. Only a few models showed outstanding (n=3, 1%) or excellent (n=32, 15%) prediction discrimination. There was a paucity (n=22, 31%) of external validation models among these developed models. Notwithstanding there were a few notable exceptions, such as FRAX with BMD (for MOF) and FRAX with BMD (for hip fracture)). Calibration of developed models (n=25, 36%) or external validation models (n=33, 24%) were rarely assessed. Moreover, no model was appraised as having a low risk of bias.

We found much variability in the geographical location of both model development and model validation. However, the majority of models were developed and validated in the UK, the US, or China. Although studies have shown that osteoporosis fractures in low or middle-income countries are also prevalent [90], no model has been developed or validated among the population from Africa, South America, and the Middle East. Tailored models for populations in these countries are important because it is well known that predictor-outcome associations vary among ethnic groups [91]. In the future, more external validation studies among the aforementioned uncovered populations are needed to improve the generalizability of existing models, which is also a cost-effective choice than investing extra research funding in developing new models [92].

Although postmenopausal females are at high risk of OF, with the increase of age, the incidence of OF in males will increase significantly. Furthermore, the mortality and disability of OF in males are higher than that in females [93]. Therefore, osteoporosis is an underestimated bone condition among the male population [94]. Although research progress has been made on OF in male [37,57], we found that most models were developed (n=31, 44%) and validated (n=76, 55%) specifically for female, with relatively less models being specifically developed (n=23, 33%) or validated (n=33, 24%) for male. Future studies are suggested to pay attention to risk prediction models specific to the male population.

It is worth noting that some models only included a few numbers of predictors (e.g., two or three predictors) [32,35,46], or easily measured predictors [29] also showed promising model performance when compared to those models [57] that used multiple complex predictors like SNPs. Moreover, due to a large number of predictors and resources demanding for measurement, the practical application of these complex models (including a large number of SNPs) is limited. On the other hand, as the gold standard for the diagnosis of osteoporosis, BMD has been included in several prediction models [34,35,39,40,46,48]. This review found that many studies showed Garvan and FRAX with BMD had higher discrimination than Garvan and FRAX without BMD [39]. However, we also observed similar or even better model performance in models without BMD, such as QFracture [84], and WHI [29], indicating that BMD may not be an essential predictor for future fracture. Hence, an increasing number of predictors or including complex predictors may not necessarily improve model performance. Complex predictors (e.g., BMD, SNPs) could be replaced by other easily measurable predictors (e.g., age, prior fractures, history of falls) for future studies under the circumstances when it is unavailable, difficult to obtain, or showed no evidence of improving model performance.

FRAX, QFracture, and Garvan are the top three commonly used models for OF prediction. FRAX (10 or 11 predictors) is a model recommended by the WHO to evaluate the risk of OF [10]. It has strong applicability and operability and has been used worldwide [17]. In this systematic review, we found that FRAX with BMD (for MOF) (n=37, 27%) was the most externally validated model, but its model performance was not particularly good; Compared with FRAX alone, the model performance of its extended model was slightly improved, but most of them had not been externally verified. The Garvan (4 predictors) contained the least predictors that are easy to measure as well [12]. That facilitates its practical use. However, the model performance of the Garvan was relatively poor [16]. The QFracture was developed through electronic medical records and showed the best model performance among the three models. Nevertheless, the larger number of predictors (26 predictors for males and 25 predictors for females) limits its practical application to a certain extent [11]. Moreover, there were some models (e.g., FRA-HS) with potentially clinical value and good performance [43], had neither been externally verified in different populations nor were rarely used in clinical practice. As a result, there is no one fit for all models being recommended in this review. The model performance, applicability, and characteristics should be considered for selecting OF prediction model [16].

Modeling methods include classical regression methods (e.g., Cox proportional hazards regression, Logistic regression) and artificial intelligence methods (e.g., machine learning). Generally, classical regression methods have the defect of lower prediction performance [57]. Compared with classical regression methods, artificial intelligence methods have a powerful ability for data analysis and exploration. Models developed through artificial intelligence methods showed the advantages of accuracy, sensitivity, and efficiency [59,95]. In this systematic review, 7 (10%) models that adopted machine learning methods indicated relatively good discrimination. However, artificial intelligence modeling requires huge and high-quality data. In addition, the model is prone to overfitting [59]. Nonetheless, with the coming of the big data era, artificial intelligence methods have more applications in the medical field and could be considered as a flexible alternative for risk prediction in large datasets.

This systematic review did not consider model impact studies, which will quantify the benefits, harms, and costs of introducing a new prediction risk model through comparative design, it is also the final crucial step to identify whether the model can be applied to the clinic [96,97]. A recent related systematic review only identified three model impact studies on OF [98]. Results from this systematic review showed that population screening could effectively reduce OF and hip fractures, however, the information on the costs and screening interval was still unclear [98]. More rigorous impact studies are needed to determine whether OF risk prediction models should be implemented in clinical practice.

### Recommendations and implications

Accurate OF risk evaluation can allow clinicians and individuals in understanding the risk of OF and guide them to make decisions to mitigate the risks [99]. When choosing a model for the prediction of OF risk, its accuracy, applicability, convenience, data availability, and cost should be considered. When developing models, simple models with less number or easily measured predictors should be considered as a priority choice to improve the clinical feasibility and practicality of the models. Given a large number of existing models, priority for the future studies should recalibrate and extend the existing OF prediction models to improve prediction performance, and conduct external verification and analysis of model impact, instead of developing new models from scratch [92].

### Strengths and limitations

The strengths of this review include systematic literature search, rigorous study selection, and detailed data extraction on the main characteristics of OF prediction models. Furthermore, we evaluated the risk of bias and applicability of all the identified models to suggest where improvements are needed in future OF prediction model studies. However, this review also has some limitations. Firstly, due to the varied heterogeneity across studies, the results were not quantitatively synthesized, which limited the comparability of models. Secondly, although we conducted an exhaustive literature search, some relevant citations may be missed due to no attempt of grey literature search. This may underestimate the number of development and validation models,

## Conclusion

In conclusion, our systematic review found that although there were a certain number of OF risk prediction models, most of the developed models had not been thoroughly internally validated or externally validated (with calibration being unassessed for most of the models). Most of the models showed poor performance as well. Moreover, all models suffered from methodological shortcomings. Given the availability of large and combined datasets, more rigorous studies are suggested to validate, improve and analyze the impact of existing OF risk prediction models in the general population rather than developing completely new models. Rigorous studies on OF prediction models are needed to target to males and the population in low or middle-income countries.

## Supplementary Materials

The Supplementary data can be found online at: www.aginganddisease.org/EN/10.14336/AD.2021.01206


