# Supplementary material for: Prediction Models for Osteoporotic Fractures Risk: A Systematic Review and Critical Appraisal
Source: Aging Dis. 2022 Jul 11;13(4):1215–38. doi: 10.14336/AD.2021.1206 (PMC9286920; doi:10.14336/AD.2021.1206)
Supplement: Supplementary file 1 [file AD-13-4-1215-s.pdf]

SUPPLEMENTARY DATA

Prediction Models for Osteoporotic Fractures Risk: A Systematic Review and Critical Appraisal

Xuemei Sun[1], Yancong Chen[1], Yinyan Gao[1], Zixuan Zhang[1], Lang Qin[1], Jinlu Song[1], Huan Wang[1], Irene XY Wu[1, 2*]

© 2021. Sun X et al. Published online at http://www.aginganddisease.org/EN/10.14336/AD.2021.1206

SUPPLEMENTARY DATA

Supplementary Table 1. Search strategy.

Pubmed (searched on April 3, 2021)

#1	"Osteoporosis"[MeSH Terms]	57154
#2	"osteoporotic fractures"[MeSH Terms]	6115
#3	"osteoporosis*"[Title/Abstract] or "osteoporotic fractures*" [Title/Abstract]	75153
#4	#1 OR #2 OR #3	93575
#5	"predictive model*"[Title/Abstract] or "prediction model*"[Title/Abstract] or "predictive rule*"[Title/Abstract] or "prediction rule*"[Title/Abstract] or "predictive equation"[Title/Abstract] or "prediction equation"[Title/Abstract] or "prediction system"[Title/Abstract]	46073
#6	"risk score*"[Title/Abstract] or "risk model*"[Title/Abstract] or "risk prediction"[Title/Abstract] or "risk calculator"[Title/Abstract] or "risk index"[Title/Abstract] or "decision model*"[Title/Abstract] or "decision rule"[Title/Abstract] or "decision curve"[Title/Abstract]	50052
#7	"machine learning"[Title/Abstract] or "artificial intelligence"[Title/Abstract] or "deep learning"[Title/Abstract] or "c statistic"[Title/Abstract] or "nomogram"[Title/Abstract] or "scoring system"[Title/Abstract] or "discrimination"[Title/Abstract] or "calibration"[Title/Abstract]	289803
#8	#5 OR #6 OR #7	362263
#9	#4 AND #8	1394

Embase (searched on April 3, 2021)

1	exp Osteoporosis/	135866
2	exp osteoporotic fractures/	19814
3	(Osteoporotic Fractures$ or Osteoporosis$).tw.	112497
4	1 or 2 or 3	164163
5	("predictive model*" or "prediction model*" or "predictive rule*" or "prediction rule*" or "predictive equation" or "prediction equation" or "prediction system").tw.	62311
6	("risk score*" or "risk model*" or "risk prediction" or "risk calculator" or "risk index" or "decision model*" or "decision rule" or "decision curve").tw.	80756
7	("machine learning" or "artificial intelligence" or "deep learning" or "c statistic" or "nomogram" or " scoring system " or "discrimination" or "calibration").tw.	356365
8	5 or 6 or 7	465906
9	4 and 8	2499
10	limit 9 to human	2274
11	limit 10 to conference abstract status	866
12	10 not 11	1408

PsycINFO (searched on April 3, 2021)

1	exp Osteoporosis/	1112
2	(Osteoporotic Fractures$ or Osteoporosis$).tw.	2140
3	1 or 2	2223
4	("predictive model*" or "prediction model*" or "predictive rule*" or "prediction rule*" or "predictive equation" or "prediction equation" or "prediction system").tw.	5594
5	("risk score*" or "risk model*" or "risk prediction" or "risk calculator" or "risk index" or "decision model*" or "decision rule" or "decision curve").tw.	6002
6	("machine learning" or "artificial intelligence" or "deep learning" or "c statistic" or "nomogram" or " scoring system " or "discrimination" or "calibration").tw.	79297
7	4 or 5 or 6	89534
8	3 and 7	35
9	limit 8 to human	34

© 2021. Sun X et al. Published online at http://www.aginganddisease.org/EN/10.14336/AD.2021.1206

SUPPLEMENTARY DATA

Supplementary Table 2. Details of the predictors included in the model.

Author	Model	Details of the predictors included in the model
Dargent- Molina 2002[25]	NR	Age, history of falls, ability to do the tandem walk, gait speed, visual acuity
Colón- Emeric 2002[26]	NR	Any: gender, ethnicity, BMI, activity of daily living difficulty, antiepileptic use, Rosow-Breslau impairment[a];<br>Hip: age, gender, ethnicity, BMI, stroke history, cognitive impairment, Rosow-Breslau impairment
McGrother 2002[27]	NR	Weight, kyphosis, poor circulation in the foot, epilepsy, corticosteroid use, poor trunk maneuver
Robbins 2007[29]	WHI	Age, general health, BMI, prior fractures, ethnicity, physical activity, smoking status, family history of fractures, corticosteroid use, treated diabetes
Albertsson 2007[28]	FRAMO	Age, weight, prior fractures, uses arms when rising 5 times from chair
Nguyen 2008[12]	Garvan	Model 1: age, femoral neck BMD, prior fractures, history of falls;<br>Model 2: age, weight, prior fractures, history of falls
Kanis 2008[10]	FRAX	Age, gender, BMI, prior fractures, family history of fractures, glucocorticoid use, smoking status, alcohol use, RA, secondary osteoporosis, femoral neck BMD
Hippisley- Cox 2009[11]	QFracture	M: age, BMI, smoking status, alcohol use, RA, cardiovascular disease, type 2 diabetes, asthma, tricyclic antidepressants use, corticosteroids use, history of falls, liver disease;<br>F: hormone replacement therapy use, age, BMI, smoking status, alcohol use, parental history of osteoporosis, RA, cardiovascular disease, type 2 diabetes, asthma, tricyclic antidepressants, corticosteroids use, history of falls, menopausal symptoms, chronic liver disease, gastrointestinal malabsorption, other endocrine disorders
Tanaka 2010[30]	FRISC	Age, weight, prior fractures, back pain, lumbar BMD
Sambrook 2011[32]	NR	Age, prior fractures
Bow 2011[33]	NR	Age, history of falls, prior fractures, physical activity, BMI, difficulty bending forward, walking aid use
Henry 2011[34]	FRISK	Hip BMD, spine BMD, history of falls, weight, prior fractures
Tamaki 2011[35]	NR	Age, weight, femoral neck BMD
Hippisley-Cox 2012[36]	Updated QFracture	F: age, BMI, ethnicity, alcohol use, smoking status, chronic obstructive pulmonary disease or asthma, any cancer, cardiovascular disease, dementia, epilepsy, history of falls, chronic liver disease, Parkinson's disease, RA or systemic lupus erythematosus, chronic renal disease, type 1 diabetes, type 2 diabetes, prior fractures, endocrine disorders, gastrointestinal malabsorption, antidepressants, corticosteroids use, unopposed hormone replacement therapy, parental history of osteoporosis;<br>M: age, BMI, ethnicity, alcohol use, smoking status, chronic obstructive pulmonary disease or asthma, any cancer, cardiovascular disease, dementia, epilepsy, history of falls, chronic liver disease, Parkinson's disease, RA or systemic lupus erythematosus, chronic renal disease, type 1 diabetes, type 2 diabetes, prior fractures, endocrine disorders, gastrointestinal malabsorption, antidepressants, corticosteroids use, unopposed hormone replacement therapy, parental history of osteoporosis, care home residence
LaFleur 2012[37]	NR	Hip:age, prior fractures, weight, complications of diabetes, malnutritive disorder, stroke history, smoking status, alcohol abuse disorder, clinic visits, history of falls;<br>MOF:age, prior fractures, weight, malnutritive disorder, opioid exposure, proton-pump inhibitor use, depression, stroke history, seizure disorder, alcohol abuse disorder, history of falls, clinic visits

Schousboe 2014[38]	NR	Age, femoral neck BMD, historical height loss, prior fractures, BMI, back pain, grip strength
Yu 2014[39]	FRAX+S	Age, gender, BMI, prior fractures, family history of fractures, glucocorticoid use, smoking status, alcohol use, RA, secondary osteoporosis, femoral neck BMD, sarcopenia
Iki 2015[40]	FRAX+TBS	Age, gender, BMI, prior fractures, family history of fractures, glucocorticoid use, smoking status, alcohol use, RA, secondary osteoporosis, femoral neck BMD, trabecular bonescore
Jang 2016[41]	NR	M: age, BMI, prior fractures, smoking status, alcohol use<br>F: age, BMI, prior fractures, family history of fractures , smoking status, RA, secondary osteoporosis
Kim 2016[42]	KFRS	Age, BMI, prior fractures, smoking status, alcohol use, physical activity, glucocorticoid use, RA, other causes of secondary osteoporosis
Francesco 2017[43]	FRA-HS	Age, gender, prior fractures, secondary osteoporosis, corticosteroids use, RA, BMI, smoking status, alcohol abuse disorder
Kruse 2017[44]	NR	M: episode of hypocholesterolemia, general practitioner consultations, phenoxymethylpenicillin use, dentist expenses, unilateral primary osteoarthritis of knee, total hip Z-Score, contusion of knee, general practitioner expenses, low-density lipoprotein cholesterol,<br>F: general practitioner expenses, episode of increased P-Glucose, samples of glucose, dentist expenses, fusidic acid, samples of thyroid stimulating hormone, dentist expenses, dentist consultation, intertrochanteric BMD, acute general practitioner srvice consultations, trochanteric bone mineral content
Li 2017[45]	NR	Unintentional weight loss, slow walking, exhaustion, weakness, physical activity
Su 2017[46]	NR	Trabecular bonescore, femoral neck BMD
Weycker 2017[47]	NR	Hip: total hip T-score , prior fractures, walking speed, mental status examination，use of arms for chair stands or poor/very poor tandem stand;<br>Non-vertebral: age, total hip T-score, history of falls, prior fractures, walking speed, Parkinson’s disease or stroke history, smoking status
Sundh 2017[48]	FRAX+MST	Age, gender, BMI, prior fractures, family history of fractures, glucocorticoid use, smoking status, alcohol use, RA, secondary osteoporosis, femoral neck BMD, mandibular sparse trabeculation
Biver 2018[50]	NR	Age, gender, BMI, prior fractures, family history of fractures, glucocorticoid use, smoking status, alcohol use, RA, secondary osteoporosis, femoral neck BMD, trabecular bonescore
Reber 2018[49]	NR	Age, gender, prior fractures
Su 2018[52]	FRAX+Fall	History of falls, age, gender, BMI, prior fractures, family history of fractures, glucocorticoid use, smoking status, alcohol use, RA, secondary osteoporosis, femoral neck BMD
Rubin 2018[51]	FREM	M: Age, schizophrenia, alcoholic liver disease, nerve root and plexus disorders, mental and behavioral disorders due to use of alcohol, other disorders of optic (2nd) nerve and visual pathways, fracture of shoulder and upper arm, pneumonia due to Streptococcus pneumoniae, fracture of forearm, hypertrophy of breast, other strabismus, fracture of rib(s), sternum and thoracic spine, vitamin B12 deficiency anemia, pleural effusion, not elsewhere classified, fracture of femur, retinal disorders in diseases classified elsewhere, dementia in Alzheimer’s disease, other symptoms and signs involving cognitive functions and awareness, secondary and unspecified malignant neoplasm of lymph nodes, symptoms and signs concerning food and fluid intake, fracture of lumbar spine and pelvis, RA, fracture of foot, except ankle, epilepsy, superficial injury of abdomen, lower back, and pelvis, fracture of lower leg, including ankle, fracture at wrist and hand

© 2021. Sun X et al. Published online at http://www.aginganddisease.org/EN/10.14336/AD.2021.1206

		level, other chronic obstructive pulmonary disease, dislocation, sprain, and strain of joints and ligaments at wrist and hand level, open wound of head, superficial injury of lower leg, superficial injury of ankle and foot, volume depletion, dislocation, sprain, and strain of joints and ligaments at ankle and foot level, osteoporosis without pathological fracture, assessments of general function level, single spontaneous delivery, abnormalities of gait and mobility, decubitus ulcer<br>F: age, chronic laryngitis and laryngotracheitis, other disorders of optic (2nd) nerve and visual pathways, alcoholic liver disease, fracture of forearm, hyperparathyroidism and other disorders of parathyroid gland, fracture of shoulder and upper arm, parkinson's disease, polyuria, mental and behavioral disorders due to use of alcohol, fracture of femur, Iridocyclitis, poisoning by psychotropic drugs, not elsewhere classified, open wound of shoulder and upper arm, fracture of lumbar spine and pelvis, other symptoms and signs involving the circulatory and respiratory systems, dental caries, fracture of lower leg, including ankle, cutaneous abscess, furuncle, and carbuncle, bacterial infection of unspecified site, osteoporosis without pathological fracture, hypotension, malignant neoplasm of prostate, fracture of rib(s), sternum, and thoracic spine, insulin-dependent diabetes mellitus, other anemias, sequelae of cerebrovascular disease, superficial injury of lower leg, superficial injury of ankle and foot, fracture at wrist and hand level, other cataract, other peripheral vascular diseases, intracranial injury, hemorrhage from respiratory passages, superficial injury of head, open wound of head, pneumonia, organism unspecified, other chronic obstructive pulmonary disease, atrial fibrillation and flutter, angina pectoris
Su 2019(1)[53]	NR	Model 1: age, total hip BMD, and femoral neck BMD<br>Model 2: age, gender, BMI, prior fractures, family history of fractures, total hip BMD, femoral neck BMD, glucocorticoid use, smoking status, alcohol use, RA, secondary osteoporosis, hip T-score
Engels 2020[54]	NR	Age, gender, prior fractures, antiparkinson, antiepileptics, aromatase inhibitors, antidiabetic agents, proton pump inhibitors, antidementives, obstructive airway diseases, Bisphosphonates, bisphosphonate combinations, raloxifene, antidepressants and psycholeptics, gestagens and estrogens, glucocorticoid, antiinflammatory and antirheumatic, calcium and vitamin D and analogues, thyreostatic, gonadotropin-releasing hormone, ophthalmic, anticholinergic and tamsulosin agents use
Kong 2020[55]	NR	Age, hip BMD, lumbar spine BMD, femoral neck BMD, arthralgia score, creatinine, homocysteine, aspartate aminotransferase, lumbar spine, trabecular bonescore, fasting glucose, triglyceride, Koreanmini-mental status examination, C-reactive protein, BMI, menarche, platelet, income status, prior fractures, thyroid-stimulating hormone, Korean geriatric depression score
Sheer 2020[56]	NR	Age, prior fractures, gender, ethnicity, history of falls, antidepressant/antipsychotic/sedative hypnotic/muscle relaxant medications use
Wu 2020[57]	NR	Age, BMI, ethnicity, alcohol use, activity of daily living difficulty, femoral neck BMD, total hip BMD, total spine BMD, ultrasound speed of sound, walking speed, mobility limitations, smoking status, 1103 Single Nucleotide Polymorphisms
Lu 2021[58]	GSOS	21,717 SNP
de Vries 2021[59]	NR	Age, gender, history of falls, prior fractures, epilepsy, frequent exposure to sunlight, total hip T-score, duration of menopause

BMD: bone mineral density; BMI: body mass index; F: female; FRA-HS: Fracture health search; FRAMO: fracture and mortality index; FRAX: fracture risk assessment tool; FREM: fracture risk evaluation model; FRISC: fracture and immobilization score; FRISK: fracture risk; gSOS: Genomic speed of sound; KFRS:Korean Fracture Risk Score; M: male; MOF: major osteoporotic fracture; MST: fracture risk assessment tool and mandibular sparse trabeculation; NR: not reported; MOF:Major osteoporotic fracture; RA: rheumatoid arthritis; SNP: Single Nucleotide Polymorphisms; TBS: trabecular bonescore; WHI: women's health initiative;
a: Rosow–Breslau impairment is defined as difficulty doing heavy work, walking upstairs, or unable to walk mile.

SUPPLEMENTARY DATA

Supplementary Table 3. Other information about the prediction models.

Author	Missing data	Treatment of continuous risk predictors	Method for selection of predictors for inclusion in multivariable modelling	Method for selection of predictors during multivariable modelling	Whether the interaction of variables is considered (Yes/No)	Model presentation
**Model development**						
Dargent-Molina 2002	Excluded	Some categorized	Univariate analysis	Stepwise	No	Risk score
Colón-Emeric 2002	NR	Some categorized	Multivariable analysis	NR	Yes	Risk score
McGrother 2002	Excluded	Some categorized	Univariate analysis	Backwards and forwards stepwise	No	Risk score
Robbins 2007	Excluded	Some categorized	Multivariable analysis	Stepwise	Yes	Risk score
Albertsson 2007	Mean imputation	Some categorized	Univariate analysis	NR	No	NR
Nguyen 2008	NR	Some categorized	Bayesian model average	Bayesian model average	No	Nomogram
Kanis 2008	NR	Some categorized	NR	Stepwise	Yes	Online calculator
Hippisley-Cox 2009	Multiple imputation	All kept continuous	Multivariable analysis	NR	Yes	Risk score
Tanaka 2010	Mean imputation	Some categorized	All candidate predictors	Backward	No	Online calculator
Yun 2010	NR	NR	NR	NR	No	Complete regression formula
Sambrook 2011	NR	Some categorized	All candidate predictors	NR	Yes	NR
Bow 2011	NR	Some categorized	All candidate predictors	Total subset	No	NR
Henry 2011	NR	NR	NR	NR	No	Risk score
Tamaki 2011	NR	NR	NR	NR	No	NR
Hippisley-Cox 2012	Multiple imputation	All kept continuous	All candidate predictors	Total subset	No	Risk score
LaFleur 2012	Excluded	Some categorized	Univariate analysis	Backward stepwise	No	Risk score
Schousboe 2014	Multiple imputation	Some categorized	Multivariable analysis	Secondary analyses	Yes	NR
Yu 2014	NR	All kept continuous	All candidate predictors	NR	No	NR
Iki 2015 (FRAX+TBS)	Excluded	All kept continuous	NR	NR	No	NR
Jang 2016	NR	All kept continuous	Univariate analysis	NR	No	Complete regression formula
Kim 2016	Excluded	Some categorized	All candidate predictors	Total subset	Yes	Risk score
Francesco 2017	Multiple imputation	Some categorized	All candidate predictors	Total subset	Yes	Complete regression formula

© 2021. Sun X et al. Published online at http://www.aginganddisease.org/EN/10.14336/AD.2021.1206

Kruse 2017	Excluded	NR	All candidate predictors	Random forest	No	NR
Li 2017	Mean imputation	All kept continuous	All candidate predictors	NR	No	Risk score
Su 2017	NR	All kept continuous	All candidate predictors	NR	No	NR
Weycker 2017	Excluded	Some categorized	Multivariable analysis	Backward and forward	Yes	Risk score
Sundh 2017	NR	Some categorized	All candidate predictors	NR	No	NR
Biver 2018 (FRAX+TBS）	NR	All kept continuous	Univariate analysis	Forward stepwise	No	NR
Reber 2018	NR	Some categorized	All candidate predictors	NR	No	Risk score
Su 2018 (FRAX+Fall)	NR	All kept continuous	All candidate predictors	Optimal subse	Yes	NR
Rubin 2018	N	Some categorized	Multistep model selection procedure	Backward	No	NR
Su 2019(1)	NR	All kept continuous	NR	NR	No	NR
Engels 2020	NR	All kept continuous	All candidate predictors	Superlearner approach	Yes	NR
Kong 2020	NR	All kept continuous	All candidate predictors	Optimal subset	No	Risk score
Sheer 2020	NR	Some categorized	Multivariable analysis	NR	No	NR
Wu 2020	NR	Some categorized	Multivariable analysis	NR	No	Risk score
Lu 2021 (gSOS)	Excluded	NA	LASSO regressions	LASSO regressions	No	NR
de Vries 2021	Multiple imputation	Some categorized	LASSO regressions	LASSO regressions	Yes	Risk score
**Model validation**						
Ensrud 2009	Excluded	All kept continuous	NA	NA	NA	NA
Hundrup 2010	Excluded	NR	NA	NA	NA	NA
Leslie 2010	NR	All kept continuous	NA	NA	NA	NA
Sornay-Rendu 2010	Excluded	All kept continuous	NA	NA	NA	NA
Trémollieres 2010	Excluded	All kept continuous	NA	NA	NA	NA
Yun 2010	NR	NR	NA	NA	NA	NA
Bolland 2011	NR	Some categorized	NA	NA	NA	NA
Langsetmo 2011	Excluded	All kept continuous	NA	NA	NA	NA
Pressman 2011	Imputation	Some categorized	NA	NA	NA	NA
Henry 2011	NR	NR	NA	NA	NA	NA
Tamaki 2011	NR	NR	NA	NA	NA	NA

© 2021. Sun X et al. Published online at http://www.aginganddisease.org/EN/10.14336/AD.2021.1206

Tanaka 2011	NR	All kept continuous	NA	NA	NA	NA
Collins 2011	Multiple imputation	All kept continuous	NA	NA	NA	NA
Fraser 2011	NR	All kept continuous	NA	NA	NA	NA
Azagra 2012	Excluded	All kept continuous	NA	NA	NA	NA
Cheung 2012	NR	All kept continuous	NA	NA	NA	NA
González-Macías 2012	Excluded	NR	NA	NA	NA	NA
Briot 2013	NR	All kept continuous	NA	NA	NA	NA
Czerwiński 2013	NR	All kept continuous	NA	NA	NA	NA
Cordomí 2013	Excluded	NR	NA	NA	NA	NA
Ettinger 2013	Excluded	All kept continuous	NA	NA	NA	NA
Rubin 2013	Excluded	Some categorized	NA	NA	NA	NA
Ahmed 2014	Excluded	NR	NA	NA	NA	NA
Friis-Holmberg 2014	Excluded	Some categorized	NA	NA	NA	NA
Van Geel 2014	NR	All kept continuous	NA	NA	NA	NA
Yu 2014	NR	All kept continuous	NA	NA	NA	NA
Klop 2016	Multiple imputation	All kept continuous	NA	NA	NA	NA
Orwoll 2017	NR	All kept continuous	NA	NA	NA	NA
Sundh 2017	NR	Some categorized	NA	NA	NA	NA
Iki 2015 (FRAX)	Excluded	All kept continuous	NA	NA	NA	NA
Dagan 2017	Multiple imputation	NR	NA	NA	NA	NA
Biver 2018 (FRAX)	NR	All kept continuous	NA	NA	NA	NA
Su 2018 (FRAX)	NR	All kept continuous	NA	NA	NA	NA
Holloway 2018	Excluded	All kept continuous	NA	NA	NA	NA
Crandall 2019	Excluded	NR	NA	NA	NA	NA
Holloway-Kew 2019	Excluded	All kept continuous	NA	NA	NA	NA
Su 2019(1)	NR	All kept continuous	NA	NA	NA	NA
Su 2019(2)	NR	All kept continuous	NA	NA	NA	NA
Tamaki 2019	NR	All kept continuous	NA	NA	NA	NA
Lu 2021 (FRAX)	Excluded	NA	NA	NA	NA	NA

EPV: events per variable; FRAX: fracture risk assessment tool; LASSO: Least absolute shrinkage and selection operator; NA: not applicable; NR: not reported; MOF: major osteoporotic fracture; TBS: trabecular bonescore

© 2021. Sun X et al. Published online at http://www.aginganddisease.org/EN/10.14336/AD.2021.1206

Supplementary Table 4. Risk of bias and applicability (using PROBAST) of included studies.

Study	ROB				Applicability			Overall	
	Participants	Predictors	Outcome	Analysis	Participants	Predictors	Outcome	ROB	Applicability
**Model development**									
Dargent-Molina 2002	+	+	-	-	+	+	-	-	-
Colón-Emeric 2002 (any）	+	+	-	-	+	+	+	-	+
Colón-Emeric 2002 (hip)	+	+	-	-	+	+	-	-	-
McGrother 2002	?	+	+	-	+	+	-	-	-
Robbins 2007	?	+	?	-	+	+	-	-	-
Albertsson 2007	?	+	+	-	+	+	-	-	-
Nguyen 2008-Model 1 (M)	?	+	?	-	+	+	+	-	+
Nguyen 2008-Model 1 (F)	?	+	?	-	+	+	+	-	+
Nguyen 2008-Model2 (M)	?	+	?	-	+	+	+	-	+
Nguyen 2008-Model 2 (F)	?	+	?	-	+	+	+	-	+
Kanis 2008 (Hip-without BMD)	+	+	+	-	+	+	-	-	-
Kanis 2008 (Hip-with BMD)	+	+	+	-	+	+	-	-	-
Kanis 2008 (MOF-without BMD)	+	+	+	-	+	+	+	-	+
Kanis 2008 (MOF-with BMD)	+	+	+	-	+	+	+	-	+
Hippisley-Cox 2009 (hip-M)	+	+	+	-	+	+	-	-	-
Hippisley-Cox 2009 (hip-F)	+	+	+	-	+	+	-	-	-
Hippisley-Cox 2009 (MOF-M)	+	+	+	-	+	+	+	-	+
Hippisley-Cox 2009 (MOF-F)	+	+	+	-	+	+	+	-	+
Tanaka 2010	+	+	?	-	+	+	+	-	+
Yun 2010 (hip）	+	?	?	-	+	+	-	-	-
Yun 2010 (MOF）	+	?	?	-	+	+	+	-	+
Sambrook 2011	+	+	?	-	+	+	-	-	-
Bow 2011	+	+	?	-	+	+	+	-	+
Henry 2011	?	+	+	-	+	+	+	-	+
Tamaki 2011 (hip）	+	+	?	-	+	+	-	-	-
Tamaki 2011 (MOF）	+	+	?	-	+	+	+	-	+

© 2021. Sun X et al. Published online at http://www.aginganddisease.org/EN/10.14336/AD.2021.1206

SUPPLEMENTARY DATA

Hippisley-Cox 2012 (hip-M)	+	+	+	-	+	+	-	-	-
Hippisley-Cox 2012 (hip-F)	+	+	+	-	+	+	-	-	-
Hippisley-Cox 2012 (MOF-M)	+	+	+	-	+	+	+	-	+
Hippisley-Cox 2012 (MOF-F)	+	+	+	-	+	+	+	-	+
LaFleur 2012 (Hip)	+	+	+	-	+	+	-	-	-
LaFleur 2012 (MOF)	+	+	+	-	+	+	+	-	+
Schousboe 2014	+	+	?	-	+	+	-	-	-
Yu 2014 (M-without BMD for hip)	+	+	?	-	+	+	-	-	-
Yu 2014 (M-with BMD for hip)	+	+	?	-	+	+	-	-	-
Yu 2014 (F-without BMD for hip)	+	+	?	-	+	+	-	-	-
Yu 2014 (F-with BMDfor hip)	+	+	?	-	+	+	-	-	-
Yu 2014 (M-without BMD for MOF)	+	+	?	-	+	+	+	-	+
Yu 2014 (M-with BMDfor MOF)	+	+	?	-	+	+	+	-	+
Yu 2014 (F-without BMD for MOF)	+	+	?	-	+	+	+	-	+
Yu 2014 (F-with BMD for MOF)	+	+	?	-	+	+	+	-	+
Iki 2015 (FRAX+TBS)	+	+	?	-	+	+	+	-	+
Jang 2016 (M)	+	+	-	-	+	+	+	-	+
Jang 2016 (F)	+	+	-	-	+	+	+	-	+
Kim 2016 (M)	-	+	+	-	+	+	+	-	+
Kim 2016 (F)	-	+	+	-	+	+	+	-	+
Francesco 2017	-	+	+	-	+	+	+	-	+
Kruse 2017 (M)	-	?	?	-	+	+	-	-	-
Kruse 2017 (F)	-	?	?	-	+	+	-	-	-
Li 2017	+	+	-	-	+	+	+	-	+
Su 2017 (F)	+	+	?	-	+	+	+	-	+
Su 2017 (F)	+	+	?	-	+	+	+	-	+
Weycker 2017 (hip)	?	?	-	-	+	+	-	-	-
Weycker 2017 (non vertebral)	?	?	-	-	+	+	-	-	-
Sundh 2017	+	+	?	-	+	+	+	-	+
Biver 2018 (FRAX, TBS）	+	+	-	-	+	+	+	-	+
Reber 2018	+	+	+	-	+	+	+	-	+
Su 2018 (FRAX+Fall-M)	?	+	?	-	+	+	+	-	+

© 2021. Sun X et al. Published online at http://www.aginganddisease.org/EN/10.14336/AD.2021.1206

Su 2018 (FRAX+Fall-F)	?	+	?	-	+	+	+	-	+
Rubin 2018 (M)	+	+	+	-	+	+	+	-	+
Rubin 2018 (F)	+	+	+	-	+	+	+	-	+
Su 2019 (1)(model 1)	?	+	+	-	+	+	+	-	+
Su 2019 (1)(model 2)	?	+	+	-	+	+	+	-	+
Engels 2020	+	+	+	-	+	+	-	-	-
Kong 2020	+	+	-	-	+	+	+	-	+
Sheer 2020	?	+	?	-	+	+	+	-	+
Wu 2020	?	+	?	-	+	+	+	-	+
Lu 2021 (gSOS for MOF)	+	+	+	-	+	+	+	-	+
Lu 2021 (gSOS for hip)	+	+	+	-	+	+	-	-	-
de Vries 2021	+	+	+	-	+	+	+	-	+
**Model validation**									
Colón-Emeric 2002 (any）	+	+	-	-	+	+	+	-	+
Colón-Emeric 2002 (hip)	+	+	-	-	+	+	-	-	-
Kanis 2008 (without BMD for hip)	+	+	+	-	+	+	-	-	-
Kanis 2008 (with BMD for hip)	+	+	+	-	+	+	-	-	-
Kanis 2008 (without BMD for MOF)	+	+	+	-	+	+	+	-	+
Kanis 2008 (with BMD for MOF)	+	+	+	-	+	+	+	-	+
Ensrud 2009 (without BMD for hip)	+	+	+	-	+	+	-	-	-
Ensrud 2009 (with BMD for hip)	+	+	+	-	+	+	-	-	-
Ensrud 2009 (without BMD for MOF)	+	+	+	-	+	+	+	-	+
Ensrud 2009 (with BMD for MOF)	+	+	+	-	+	+	+	-	+
Tanaka 2010	+	+	?	-	+	+	+	-	+
Hundrup 2010	+	+	+	-	+	+	-	-	-
Leslie 2010 (without BMD for hip)	+	+	+	-	+	+	-	-	-
Leslie 2010 (with BMD for hip)	+	+	+	-	+	+	-	-	-
Leslie 2010 (without BMD for MOF)	+	+	+	-	+	+	+	-	+
Leslie 2010 (with BMD for MOF)	+	+	+	-	+	+	+	-	+
Sornay-Rendu 2010(without BMD for MOF)	?	+	+	-	+	+	+	-	+
Sornay-Rendu 2010(with BMD for MOF)	?	+	+	-	+	+	+	-	+

© 2021. Sun X et al. Published online at http://www.aginganddisease.org/EN/10.14336/AD.2021.1206

SUPPLEMENTARY DATA

Trémollieres 2010	?	+	+	-	+	+	+	-	+
Yun 2010 (FRAX for hip）	+	?	?	-	+	+	-	-	-
Yun 2010 (FRAX for MOF）	+	?	?	-	+	+	+	-	+
Bolland 2011 (Garvan for hip）	+	?	-	-	+	+	-	-	-
Bolland 2011 (Garvan for MOF ）	+	?	-	-	+	+	+	-	+
Bolland 2011 (FRAX without BMD for hip）	+	?	-	-	+	+	-	-	-
Bolland 2011 (FRAX with BMD for hip）	+	?	-	-	+	+	-	-	-
Bolland 2011 (FRAX without BMD for MOF)	+	?	-	-	+	+	+	-	+
Bolland 2011 (FRAX with BMDfor MOF)	+	?	-	-	+	+	+	-	+
Langsetmo 2011 (hip-M)	?	+	+	-	+	+	-	-	-
Langsetmo 2011 (hip-F)	?	+	+	-	+	+	-	-	-
Langsetmo 2011 (MOF-M)	?	+	+	-	+	+	+	-	+
Langsetmo 2011 (MOF-F)	?	+	+	-	+	+	+	-	+
Pressman 2011 (without BMD)	+	+	+	-	+	+	-	-	-
Pressman 2011 (with BMD)	+	+	+	-	+	+	-	-	-
Henry 2011 (FRAX without BMD)	?	+	+	-	+	+	+	-	+
Henry 2011 (FRAX with BMD)	?	+	+	-	+	+	+	-	+
Henry 2011 (Garvan)	?	+	+	-	+	+	+	-	+
Tamaki 2011 (FRAX without BMD for hip)	+	+	?	-	+	+	-	-	-
Tamaki 2011 (FRAXwith BMD for hip)	+	+	?	-	+	+	-	-	-
Tamaki 2011 (FRAX without BMD for MOF)	+	+	?	-	+	+	+	-	+
Tamaki 2011 (FRAX with BMD for MOF)	+	+	?	-	+	+	+	-	+
Tanaka 2011	+	+	+	-	+	+	+	-	+
Collins 2011 (MOF-M)	+	+	+	?	+	+	+	?	+
Collins 2011 (MOF-F)	+	+	+	?	+	+	+	?	+
Collins 2011 (M for hip)	+	+	+	?	+	+	-	?	-
Collins 2011 (F for hip)	+	+	+	?	+	+	-	?	-
Fraser 2011 (MOF without BMD)	+	+	?	-	+	+	+	-	+
Fraser 2011 (MOF with BMD)	+	+	?	-	+	+	+	-	+
Fraser 2011 (without BMD for hip)	+	+	?	-	+	+	-	-	-
Fraser 2011 (with BMD for hip)	+	+	?	-	+	+	-	-	-

© 2021. Sun X et al. Published online at http://www.aginganddisease.org/EN/10.14336/AD.2021.1206

SUPPLEMENTARY DATA

Azagra 2012 (without BMD for MOF)	+	+	?	-	+	+	+	-	+
Azagra 2012 (with BMD for MOF)	+	+	?	-	+	+	+	-	+
Azagra 2012 (without BMD for hip)	+	+	?	-	+	+	-	-	-
Azagra 2012 (with BMD for hip)	+	+	?	-	+	+	-	-	-
Cheung 2012 (without BMD for MOF)	+	+	?	-	+	+	+	-	+
Cheung 2012 (with BMD for MOF)	+	+	?	-	+	+	+	-	+
Cheung 2012 (without BMD for hip)	+	+	?	-	+	+	-	-	-
Cheung 2012 (with BMD for hip)	+	+	?	-	+	+	-	-	-
González-Macías 2012 (hip)	+	+	+	-	+	+	-	-	-
González-Macías 2012 (MOF)	+	+	+	-	+	+	+	-	+
Briot 2013 (without BMD)	+	+	+	-	+	+	+	-	+
Briot 2013 (with BMD)	+	+	+	-	+	+	+	-	+
Czerwiński 2013	+	+	-	-	+	+	+	-	+
Cordomí 2013	+	+	-	-	+	+	+	-	+
Ettinger 2013 (without BMD for MOF)	+	+	+	-	+	+	+	-	+
Ettinger 2013 (with BMD for MOF)	+	+	+	-	+	+	+	-	+
Ettinger 2013 (without BMD for hip)	+	+	+	-	+	+	-	-	-
Ettinger 2013 (with BMD for hip)	+	+	+	-	+	+	-	-	-
Rubin 2013	+	+	+	-	+	+	+	-	+
Ahmed 2014 (model 1-M)	+	+	?	-	+	+	+	-	+
Ahmed 2014 (model 1-F)	+	+	?	-	+	+	+	-	+
Ahmed 2014 (model 2-M)	+	+	?	-	+	+	+	-	+
Ahmed 2014 (model 2-F)	+	+	?	-	+	+	+	-	+
Friis-Holmberg 2014 (M for hip)	+	+	+	-	+	+	-	-	-
Friis-Holmberg 2014 (F for hip)	+	+	+	-	+	+	-	-	-
Friis-Holmberg 2014 (M for MOF)	+	+	+	-	+	+	+	-	+
Friis-Holmberg 2014 (F for MOF)	+	+	+	-	+	+	+	-	+
Van Geel 2014 ((FRAX for hip)	+	+	?	-	+	+	-	-	-
Van Geel 2014 ((FRAX without BMD for MOF)	+	+	?	-	+	+	+	-	+
Van Geel 2014 ((FRAX with BMD for MOF)	+	+	?	-	+	+	+	-	+
Van Geel 2014 (Garvan model 1 for hip)	+	+	?	-	+	+	-	-	-

© 2021. Sun X et al. Published online at http://www.aginganddisease.org/EN/10.14336/AD.2021.1206

SUPPLEMENTARY DATA

Van Geel 2014 (Garvan model 1 for MOF)	+	+	?	-	+	+	+	-	+
Van Geel 2014 (Garvan model 2 for hip)	+	+	?	-	+	+	-	-	-
Van Geel 2014 (Garvan model 2 for MOF)	+	+	?	-	+	+	+	-	+
Yu 2014 (FRAX without BMDfor hip-M)	+	+	?	-	+	+	-	-	-
Yu 2014 (FRAX with BMD for hip-M)	+	+	?	-	+	+	-	-	-
Yu 2014 (FRAXwithout BMD for hip-F)	+	+	?	-	+	+	-	-	-
Yu 2014 (FRAXwith BMD for hip-F)	+	+	?	-	+	+	-	-	-
Yu 2014 (FRAX without BMD for MOF-M)	+	+	?	-	+	+	+	-	+
Yu 2014 (FRAX with BMD for MOF)	+	+	?	-	+	+	+	-	+
Yu 2014 (FRAX without BMD for MOF-F)	+	+	?	-	+	+	+	-	+
Yu 2014 (FRAX with BMD for MOF-F)	+	+	?	-	+	+	+	-	+
Klop 2016 (hip)	+	+	+	?	+	+	-	?	-
Klop 2016 (MOF)	+	+	+	?	+	+	+	?	+
Orwoll 2017 (Hip-Sweden)	+	+	?	-	+	+	-	-	-
Orwoll 2017 (Hip-US)	+	+	?	-	+	+	-	-	-
Orwoll 2017 (Hip-China)	+	+	?	-	+	+	-	-	-
Orwoll 2017 (MOF-Sweden)	+	+	?	-	+	+	+	-	+
Orwoll 2017 (MOF-US)	+	+	?	-	+	+	+	-	+
Orwoll 2017 (MOF-China)	+	+	?	-	+	+	+	-	+
Sundh 2017	+	+	?	-	+	+	+	-	+
Iki 2015 (FRAX)	+	+	?	-	+	+	+	-	+
Francesco 2017	?	+	+	-	+	+	+	-	+
Dagan 2017 (QFracture for MOF)	+	+	+	?	+	+	+	?	+
Dagan 2017 (QFracture for hip)	+	+	+	?	+	+	-	?	-
Dagan 2017 (FRAX for MOF)	+	+	+	?	+	+	+	?	+
Dagan 2017 (FRAX for hip)	+	+	+	?	+	+	-	?	-
Dagan 2017 (Garvan for MOF)	+	+	+	?	+	+	+	?	+
Dagan 2017 (Garvan for hip)	+	+	+	?	+	+	-	?	-

© 2021. Sun X et al. Published online at http://www.aginganddisease.org/EN/10.14336/AD.2021.1206

SUPPLEMENTARY DATA

Biver 2018 (FRAX)	+	+	-	-	+	+	+	-	+
Su 2018(FRAX-M)	?	+	?	-	+	+	+	-	+
Su 2018(FRAX-F)	?	+	?	-	+	+	+	-	+
Holloway 2018 (hip）	+	+	?	-	+	+	-	-	-
Holloway 2018 (MOF）	+	+	?	-	+	+	+	-	+
Crandall 2019 (FRAX for hip)	+	+	-	-	+	+	-	-	-
Crandall 2019 (FRAX for MOF)	+	+	-	-	+	+	+	-	+
Crandall 2019 (Garvan for hip)	+	+	-	-	+	+	-	-	-
Crandall 2019 (Garvan for MOF)	+	+	-	-	+	+	+	-	+
Holloway-Kew 2019 (FRAX without BMD-F)	+	+	+	-	+	+	+	-	+
Holloway-Kew 2019 (FRAX with BMD-F)	+	+	+	-	+	+	+	-	+
Holloway-Kew 2019 (FRAX without BMD-M)	+	+	+	-	+	+	+	-	+
Holloway-Kew 2019 (FRAX with BMD-M)	+	+	+	-	+	+	+	-	+
Holloway-Kew 2019 (Garvan model 1-M)	+	+	+	-	+	+	+	-	+
Holloway-Kew 2019 (Garvan model 1-F)	+	+	+	-	+	+	+	-	+
Holloway-Kew 2019 (Garvan model 2-M)	+	+	+	-	+	+	+	-	+
Holloway-Kew 2019 (Garvan model 2-F)	+	+	+	-	+	+	+	-	+
Su 2019 (1)	?	+	+	-	+	+	+	-	+
Su 2019 (2)(FRAX+TBS-M)	?	+	?	-	+	+	+	-	+
Su 2019 (2)(FRAX+TBS-F)	?	+	?	-	+	+	+	-	+
Su 2019 (2)(FRAX-M)	?	+	?	-	+	+	+	-	+
Su 2019 (2)(FRAX-F)	?	+	?	-	+	+	+	-	+
Tamaki 2019 (FRAX+TBS-without BMD)	+	+	?	-	+	+	+	-	+
Tamaki 2019 (FRAX+TBS with BMD)	+	+	?	-	+	+	+	-	+
Tamaki 2019 (FRAX without BMD)	+	+	?	-	+	+	+	-	+
Tamaki 2019 (FRAX with BMD)	+	+	?	-	+	+	+	-	+
Lu 2021 (gSOS for MOF)	+	+	+	-	+	+	+	-	+

© 2021. Sun X et al. Published online at http://www.aginganddisease.org/EN/10.14336/AD.2021.1206

Lu 2021 (gSOS for hip)	+	+	+	-	+	+	-	-	-
Lu 2021 (FRAX for MOF)	+	+	+	-	+	+	+	-	+
Lu 2021 (FRAX for hip)	+	+	+	-	+	+	-	-	-

F: female; FRAX: fracture risk assessment tool; gSOS: genomic speed of sound; M: male; MOF: major osteoporotic fracture; PROBAST: Prediction model Risk Of Bias ASsessment Tool; ROB: risk of bias; TBS: trabecular bone score; +: low risk of bias//low concern regarding applicability; ?: unclear risk of bias/low concern regarding applicability; -: high risk of bias/high concern regarding applicability.

© 2021. Sun X et al. Published online at http://www.aginganddisease.org/EN/10.14336/AD.2021.1206
